# Nociceptive transient receptor potential canonical 7 (TRPC7) mediates aging‐associated tumorigenesis induced by ultraviolet B

**DOI:** 10.1111/acel.13075

**Published:** 2019-11-21

**Authors:** Wen‐Li Hsu, Ming‐Hsien Tsai, Ching‐Ying Wu, Jui‐Lin Liang, Jian‐He Lu, Jennifer S. Kahle, Hsin‐Su Yu, Chia‐Jung Yen, Chen‐Tung Yen, Yi‐Chun Hsieh, Yung‐Yun Huang, Li‐Ching Lin, Tsung‐Fu Tsai, Chu‐Huang Chen, Tohru Yoshioka

**Affiliations:** ^1^ Research Organization for Nano & Life Innovation Waseda University, Shinjuku Tokyo Japan; ^2^ Emerging Compounds Research Center General Research Service Center National Pingtung University of Science and Technology Pingtung Taiwan; ^3^ Regenerative Medicine and Cell Therapy Research Center Kaohsiung Medical University Kaohsiung Taiwan; ^4^ Department of Child Care College of Humanities and Social Sciences National Pingtung University of Science and Technology Pingtung Taiwan; ^5^ Department of Dermatology Kaohsiung Municipal Ta‐Tung Hospital Kaohsiung Medical University Hospital Kaohsiung Medical University Kaohsiung Taiwan; ^6^ Graduate Institute of Medicine School of Medicine Kaohsiung Medical University Kaohsiung Taiwan; ^7^ Department of General Surgery Chi‐Mei Medical Center, Liouying Tainan Taiwan; ^8^ Department of Dermatology Kaohsiung Medical University Kaohsiung Taiwan; ^9^ Department of Psychological Sciences University of San Diego San Diego CA USA; ^10^ BPS, International San Diego CA USA; ^11^ Department of Life Science National Taiwan University Taipei Taiwan; ^12^ School of Medicine Kaohsiung Medical University Kaohsiung Taiwan; ^13^ Center for Lipid Biosciences Kaohsiung Medical University Hospital Kaohsiung Medical University Kaohsiung Taiwan; ^14^ Vascular and Medicinal Research Texas Heart Institute Houston TX USA; ^15^ New York Heart Research Foundation Mineola NY USA; ^16^ Lipid Science and Aging Research Center Kaohsiung Medical University Kaohsiung Taiwan

**Keywords:** aging, p53, TRPC7, tumor initiator gene, tumorigenesis, ultraviolet pathology

## Abstract

Aging, cancer, and longevity have been linked to intracellular Ca^2+^ signaling and nociceptive transient receptor potential (TRP) channels. We found that TRP canonical 7 (TRPC7) is a nociceptive mechanoreceptor and that TRPC7 channels specifically mediate the initiation of ultraviolet B (UVB)‐induced skin aging and tumor development due to p53 gene family mutations. Within 30 min after UVB irradiation, TRPC7 mediated UVB‐induced Ca^2+^ influx and the subsequent production of reactive oxygen species in skin cells. Notably, this function was unique to TRPC7 and was not observed for other TRP channels. In *TRPC7* knockout mice, we did not observe the significant UVB‐associated pathology seen in wild‐type mice, including epidermal thickening, abnormal keratinocyte differentiation, and DNA damage response activation. *TRPC7* knockout mice also had significantly fewer UVB‐induced cancerous tumors than did wild‐type mice, and UVB‐induced p53 gene family mutations were prevented in *TRPC7* knockout mice. These results indicate that TRPC7 activity is pivotal in the initiation of UVB‐induced skin aging and tumorigenesis and that the reduction in TRPC7 activity suppresses the UVB‐induced aging process and tumor development. Our findings support that *TRPC7* is a potential tumor initiator gene and that it causes cell aging and genomic instability, followed by a change in the activity of proto‐oncogenes and tumor suppressor genes to promote tumorigenesis.

## Graphical abstract

Schematic representation of the role of TRPC7 as a potential tumor initiator gene in ultraviolet B (UVB)‐induced aging and cancer progression. UVB‐activated TRPC7 initiates skin aging via intracellular Ca^2+^ elevation, resulting in oxidative stress, DNA damage response activation, abnormal differentiation, and senescence inflammation response activation; this pathology is repaired with the activation of p53 to maintain tissue homeostasis. When homeostasis is no longer maintained and the aging process is activated, carcinogens promote cancer progression. TRPC7 initiates tumorigenesis by causing genomic instability, thereby changing the activity of proto‐oncogenes and tumor suppressor genes.

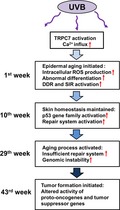

## 1 INTRODUCTION

An association between transient receptor potential (TRP) channels and age‐related diseases (e.g., cancer, Alzheimer's disease, cardiovascular disease) has been well documented (Santoni & Farfariello, 2011; Takada, Numata, & Mori, 2013; Yue et al., 2015); however, the specific roles of TRP channels in aging and the development of these diseases remain unclear. In a recent study, capsaicin‐sensitive nociceptor TRP vanilloid 1 (TRPV1) channel activity in the peripheral sensory neurons of both mice and nematodes was shown to affect longevity and overall metabolism through Ca^2+^‐dependent neuroendocrine regulation, such as the dorsal root ganglion innervation of pancreatic cells (Riera et al., 2014). In addition, *TRPV1* knockout mice exhibited pain insensitivity and increased longevity similar to those of naked mole‐rats (Buffenstein, 2008; Park et al., 2008; Seluanov et al., 2009), which usually live for an astonishing 28 years. Naked mole‐rats distinctly lack TRP pain receptors and are primarily youthful and healthy (Buffenstein, 2008), qualities that are attributable to their characteristic pain insensitivity (Park et al., 2008), cancer resistance (Seluanov et al., 2009), low respiratory rate, flexible glycolytic metabolism, and low production of reactive oxygen species (ROS; Park et al., 2017). A key link between the longevity of *TRPV1* knockout mice and that of naked mole‐rats is that nerve growth factor does not sensitize TRPV1 channels in naked mole‐rats; furthermore, because Ca^2+^ influx is reduced in naked mole‐rats, inflammatory heat hyperalgesia is not induced (Omerbasic et al., 2016).

Recently, we showed that TRPC7 is involved in ultraviolet B (UVB)‐induced skin aging and diacylglycerol production in the cytoplasmic membrane and that this skin aging could be prevented by attenuating the UVB‐induced elevation of intracellular Ca^2+^ concentration ([Ca^2+^]_i_; Hsu et al., 2015). The only other known physiologic function of TRPC7 reported to date is its role in the initiation of seizure activity (Phelan, Shwe, Abramowitz, Birnbaumer, & Zheng, 2014). Thus, TRPC7 has been implicated in several pathologic processes, but little is known about TRPC7’s specificity of function or the underlying mechanisms. In this study, we sought to comprehensively examine TRPC7 function in the skin. We first characterized TRPC7 as a nociceptive mechanoreceptor; then, we performed a screen in human keratinocytes to determine that, among other TRP channels, TRPC7 is uniquely involved in UVB‐induced skin aging. Furthermore, we identified an early time frame of UVB‐induced TRPC7 activation and characterized its downstream effects on skin pathology over time, as well as the mechanisms underlying the effects of UVB‐induced TRPC7 activation. Importantly, we show for the first time to our knowledge that TRPC7 plays a critical role in all steps of tumorigenesis, starting with the initiation of tumor formation.

## 2 RESULTS

### 2.1 UVB‐induced Ca^2+^ elevation is due to nociceptive mechanoreceptor TRPC7 in keratinocytes

**Figure 1:**
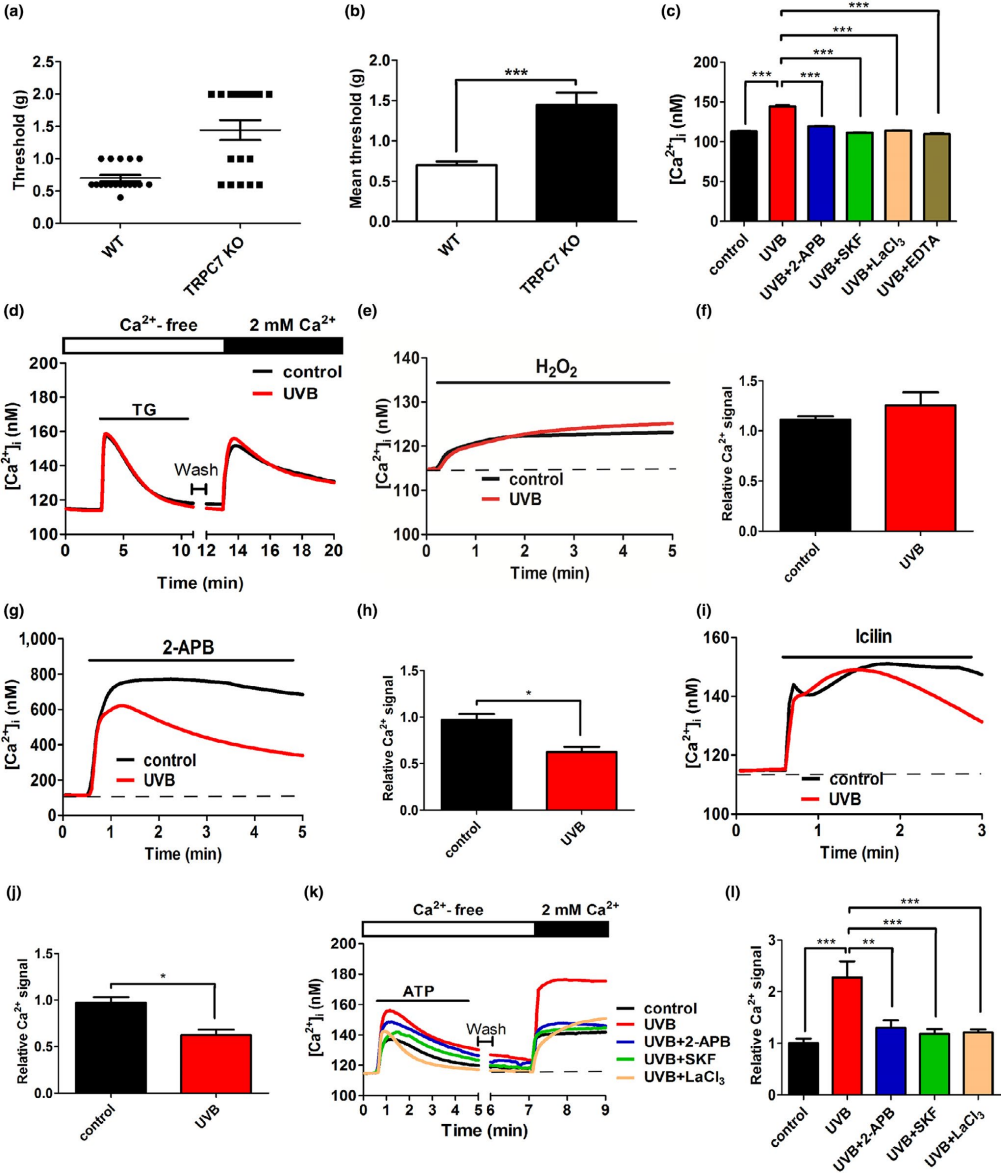
Physiologic role of TRPC7 as a nociceptive mechanoreceptor and the specificity of its cellular mechanism to the TRPC family. (a) Calibrated von Frey filaments were used to assess mechanical hyperalgesia in wild‐type (WT, *n* = 5) and *TRPC7^−/−^* knockout mice (KO, *n* = 4). The threshold grams of force that elicited a response in WT and *TRPC7^−/−^* knockout mice were recorded and averaged. (b) The mean (± *SD*) thresholds were significantly different between wild‐type and *TRPC7^−/−^* knockout mice. (c) TRPC7 initiated UVB‐induced [Ca^2+^]_i_ elevation in human primary keratinocytes. The mean (± *SD*) [Ca^2+^]_i_ from 195 keratinocytes with or without UVB irradiation after a 30‐min pretreatment with 1 mM EDTA (extracellular Ca^2+^ chelator) or a TRPC inhibitor (50 μM 2‐APB, 25 μM SKF96365 [SKF], or 100 μM LaCl_3_). (d) UVB irradiation did not affect thapsigargin (TG)‐induced store‐operated Ca^2+^ mobilization or Ca^2+^ store depletion‐induced Ca^2+^ influx in keratinocytes. Intracellular Ca^2+^ responses were measured by using Ca^2+^ imaging after treating keratinocytes with (e) hydrogen peroxide (H_2_O_2_; mean ± *SD*, *n* = 285 cells [f]), (g) 2‐aminoethoxydiphenyl borate (2‐APB; mean ± *SD*, *n* = 285 cells [h]), or (i) icilin (mean ± *SD*, *n* = 285 cells [j]). (k) After the application of ATP to activate TRPCs via the phospholipase C (PLC) pathway in Ca^2+^‐free balanced salt solution, extracellular CaCl_2_ was added. (l) The mean (± *SD*) area under the [Ca^2+^]_i_ response curves from 250 keratinocytes after the addition of extracellular CaCl_2_. The duration of application is indicated by thin black bars. Student's *t* test: *p < .05, **p < .01, ***p < .001

We first characterized the physiologic role of TRPC7 in the skin by using *TRPC7^−/−^* knockout mice and found that TRPC7 is a novel mechanoreceptor that transduces mechanical pressure into pain signals (Figure 1a,b, and S1). Next, we examined the mechanism underlying the involvement of TRPC7 in skin aging by testing our hypothesis that TRPC7 initiates UVB‐induced skin aging via [Ca^2+^]_i_ elevation. To determine whether TRPC7 is the specific TRP channel involved in UVB‐induced [Ca^2+^]_i_ elevation and the subsequent production of ROS, we performed experiments with known UVB‐activated TRP channel agonists and antagonists (Table S1) in cultured human keratinocytes. UVB‐induced [Ca^2+^]_i_ elevation was attenuated by the pretreatment of keratinocytes with 2‐aminoethoxydiphenyl borate (2‐APB), SKF96365, or LaCl_3_, all of which are TRPC inhibitors (Figure 1c). We then tested the involvement of other TRP channels in the skin (Toth, Olah, Szollosi, & Biro, 2014), including TRPVs, TRP melastatin (TRPMs), or TRP ankyrin 1 (TRPA1). Ca^2+^ influx induced by Ca^2+^ store depletion was not markedly different in the presence or absence of UVB pre‐exposure (Figure 1d), indicating that TRPV6 and TRPM2 channels are not involved in UVB‐induced [Ca^2+^]_i_ elevation. Similarly, H_2_O_2_‐induced [Ca^2+^]_i_ elevation was not significantly different in the presence or absence of UVB pre‐exposure, also indicating that TRPM2 channels are not involved in UVB‐induced [Ca^2+^]_i_ elevation (Figure 1e,f). Additionally, TRPV1 and TRPV3 did not initiate UVB‐induced [Ca^2+^]_i_ elevation because the Ca^2+^ response to 2‐APB (an agonist of TRPV1 and TRPV3; Table S1) was inhibited after UVB exposure (Figure 1g,h). Similarly, the Ca^2+^ response to icilin was inhibited after UVB irradiation, indicating that neither TRPM8 nor TRPA1 is involved in UVB‐induced [Ca^2+^]_i_ elevation (Figure 1i,j). Together, these results indicate that TRPC channels specifically are important in the initial stages of UVB‐induced [Ca^2+^]_i_ elevation. Consistent with this, adenosine triphosphate (ATP)‐induced Ca^2+^ mobilization via the phospholipase C pathway resulted in the greatest subsequent influx of extracellular Ca^2+^ after UVB irradiation, and this effect was blocked by the pretreatment of keratinocytes with the TRPC inhibitors 2‐APB, SKF96365, or LaCl_3_ (Figure 1k,l).

### 2.2 UVB‐induced ROS production specifically results from TRPC7‐mediated Ca^2+^ influx

**Figure 2:**
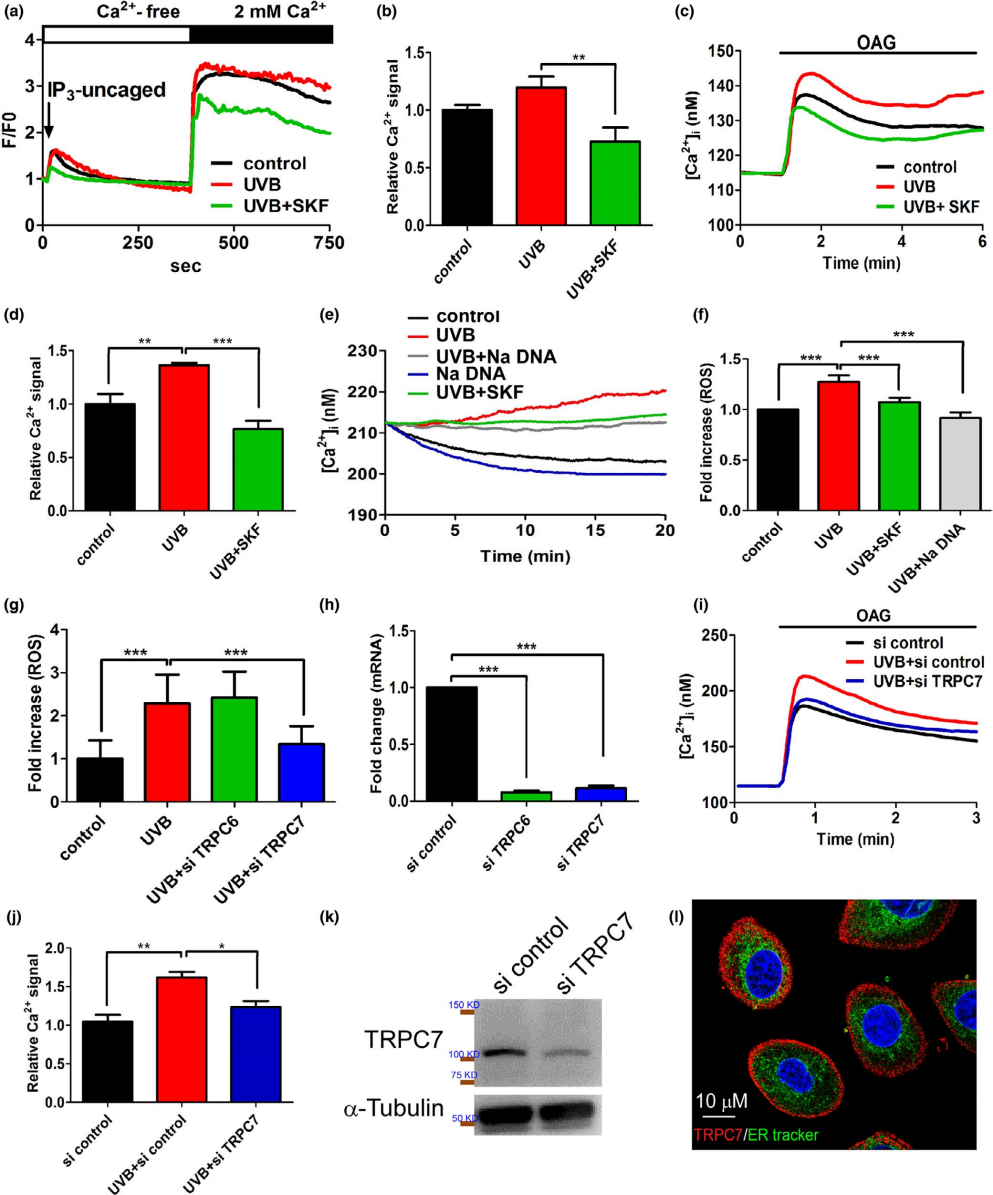
Ultraviolet B (UVB)‐induced [Ca^2+^] elevation in keratinocytes mediated specifically by Ca^2+^ influx via TRPC7. (a) UVB exposure had no effect on uncaged inositol trisphosphate (IP_3_)‐induced activation of TRPC1, TRPC4, and TRPC5 or the subsequent [Ca^2+^]_i_ elevation in keratinocytes. SKF96365 pretreatment (positive control) significantly decreased UVB‐induced [Ca^2+^]_i_ elevation. (b) The mean (±*SD*) area under the [Ca^2+^]_i_ response curves from 60 cells after the addition of extracellular CaCl_2_. (c) In contrast, UVB pre‐exposure significantly increased [Ca^2+^]_i_ elevation in response to 1‐oleoyl‐2‐acetyl‐sn‐glycerol (OAG), an effect that was significantly inhibited by SKF pretreatment. (d) The mean (± *SD*) area under the [Ca^2+^]_i_ response curves from 235 cells after the addition of OAG. The knockdown of *TRPC7* significantly decreased UVB‐induced cell damage via the reduction in the p53 pathway activation. UVB‐induced Ca^2+^ elevation via TRPC7 channels initiated intracellular reactive oxygen species (ROS) production in keratinocytes. (e) The time course of the increased Ca^2+^ response after UVB exposure demonstrated an immediate UVB‐induced increase in the intracellular Ca^2+^ concentration that was inhibited by pretreatment with the TRPC7 inhibitors SKF96365 (SKF) or Na DNA. (f) Intracellular ROS production began after 30 min of UVB exposure. Cells were stained with 5 μM dihydroethidium (free radical indicator), and the intensity of emitted fluorescence was analyzed by using an Olympus fluorescence microscope (*n* > 200 cells; mean ± *SD*). (g) Intracellular ROS production increased after UVB irradiation (*n* > 200 cells; mean ± *SD*). This effect was significantly reduced by the knockdown of *TRPC7*, but not the knockdown of *TRPC6*. (h) The knockdown of *TRPC6* and *TRPC7* mRNA expression was effective. (i) *TRPC7* knockdown attenuated the OAG‐induced increase in [Ca^2+^]_i_ after UVB exposure. (j) The mean area under the [Ca^2+^]_i_ response curves from 260 cells after OAG application. (k) Western blot analysis showing that protein expression was reduced after *TRPC7* knockdown in keratinocytes. (l) Immunofluorescence staining showing the localization of TRPC7 (red) to the cell membrane of keratinocytes. The endoplasmic reticulum is green (ER‐Tracker; 1,3,5,7‐tetramethyl‐8‐phenyl‐4,4‐difluoroboradiazaindacene; BODIPY), and nuclei are blue (4′,6‐diamidino‐2‐phenylindole; DAPI). si, small interfering RNAs. *p < .05; **p < .01; ***p < .001

We then narrowed down which TRPC(s) in the skin initiate UVB‐induced [Ca^2+^]_i_ elevation via the phospholipase C pathway. IP_3_ activates TRPC1, TRPC4, and TRPC5 channels, whereas diacylglycerol activates TRPC6 and TRPC7 channels (Harteneck & Leuner, 2014). No difference was observed in uncaged IP_3_–mediated Ca^2+^ influx in keratinocytes in the presence or absence of UVB pre‐exposure (Figure 2a,b). However, UVB pre‐exposure induced an increase in Ca^2+^ influx in the presence of the diacylglycerol analogue 1‐oleoyl‐2‐acetyl‐sn‐glycerol (OAG) (Figure 2c,d), an effect that was inhibited by SKF96365. These results indicate that the channels most likely involved in UVB‐induced [Ca^2+^]_i_ elevation are TRPC6 and TRPC7.

UVB‐induced [Ca^2+^]_i_ elevation triggers the generation of intracellular ROS (Masaki, Izutsu, Yahagi, & Okano, 2009). We found that UVB‐induced [Ca^2+^]_i_ elevation and ROS production began within 30 min after UVB irradiation but were inhibited by TRPC inhibitors in keratinocytes (Figure 2e,f). Furthermore, the knockdown of *TRPC7* but not *TRPC6* significantly inhibited UVB‐induced ROS production in keratinocytes (Figure 2g,h). Moreover, the knockdown of *TRPC7* significantly decreased Ca^2+^ influx induced by the diacylglycerol analogue OAG after UVB irradiation (Figure 2i–k). We further showed that TRPC7 was localized to the plasma membrane in keratinocytes (Figure 2l). These results indicate that UVB‐induced [Ca^2+^]_i_ elevation results from the influx of extracellular Ca^2+^ and not the mobilization of Ca^2+^ from intracellular stores, and that this specifically occurs through TRPC7 channels.

### 2.3 TRPC7 mediates UVB‐induced epidermal pathology in mice

**Figure 3:**
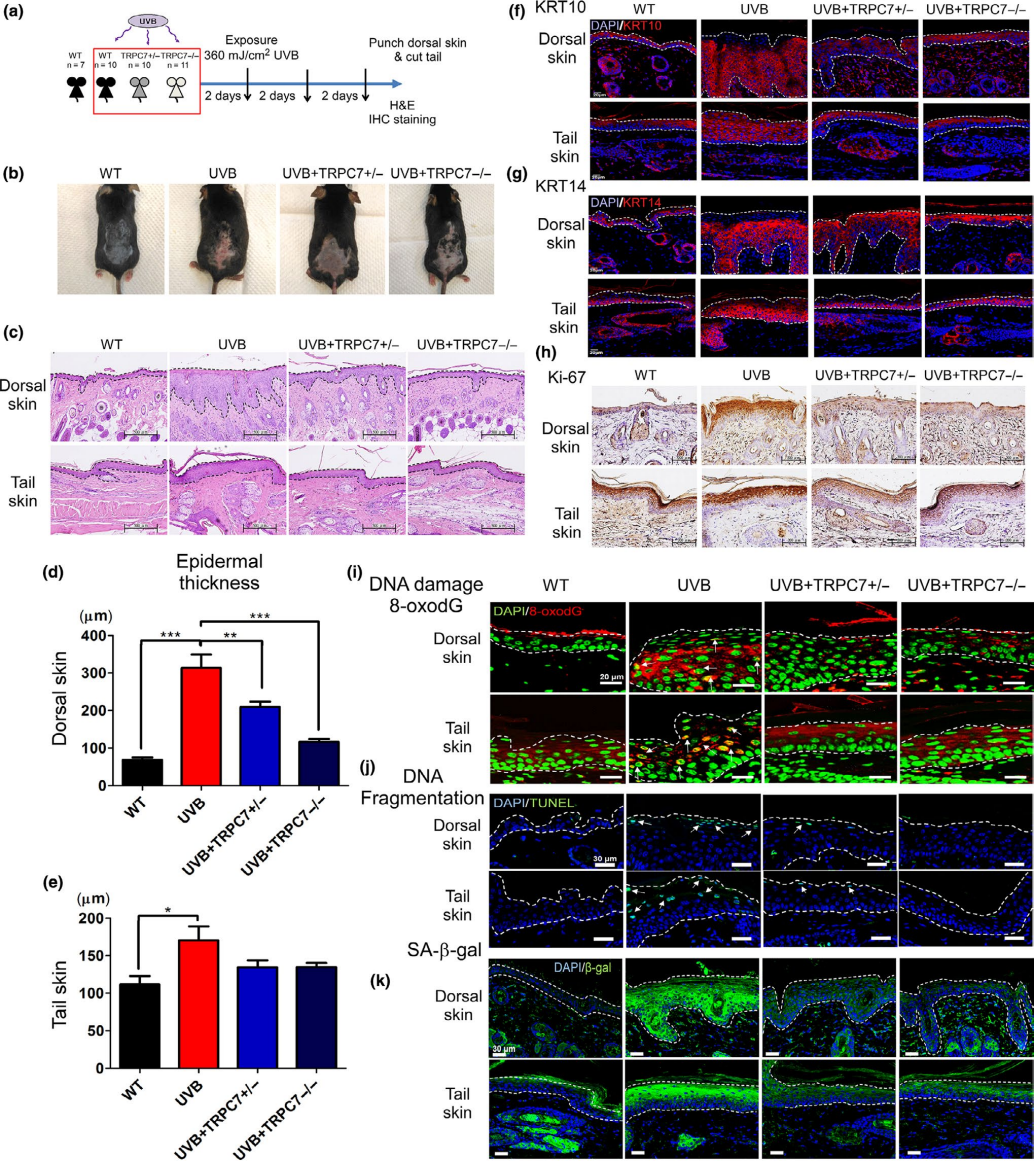
Role of ultraviolet B (UVB)‐induced [Ca^2+^]_i_ elevation via TRPC7 in initiating epidermal aging, abnormal differentiation, and cell senescence through oxidative stress and DNA damage response activation. (a) A schematic showing the experimental design (wild‐type [WT], *n* = 7; UVB, *n* = 10; *TRPC7^+/−^*, *n* = 10 and *TRPC7^−/−^*, *n* = 11). Mice were exposed to UVB three times weekly. Dorsal skin and tail skin punches or clippings were sectioned for histologic and immunohistochemical analysis. (b) Photographs showing the skin's appearance. (c) Hematoxylin‐and‐eosin–stained images of dorsal skin and tail skin. Dotted lines indicate the boundaries of the epidermis. Quantification of the mean (± *SD*) epidermal thickness in (d) dorsal skin and (e) tail skin. *p < .05; **p < .01; ***p < .001. Differentiating cells were detected by using (f) keratin 10 (KRT10; keratinocytes) and (g) keratin 14 (KRT14; epidermis). (h) Ki‐67 expression is shown, indicating proliferating epidermal cells (brown, counterstained with hematoxylin). Activation of the DNA damage response was analyzed by using (i) 8‐oxo‐2′‐deoxyguanosine (8‐oxodG) staining and (j) terminal deoxynucleotidyl transferase dUTP nick end labeling (TUNEL) staining. White arrows indicate cells with DNA damage. (k) Cell senescence was determined by detecting senescence‐associated β‐galactosidase (SA‐β‐gal) activity. SA‐β‐gal activity was reduced in the skin of *TRPC7* knockout mice

Next, we performed experiments using *TRPC7^+/−^* and *TRPC7^−/−^* knockout mice to determine whether UVB‐induced [Ca^2+^]_i_ elevation via TRPC7 initiates cell senescence through oxidative stress and activation of the DNA damage response (DDR), leading to epidermal aging. In the absence of UVB, skin phenotype was similar between wild‐type and *TRPC7^+/−^* or *TRPC7^−/−^* mice (Figure S2). In wild‐type mice, UVB exposure induced severe desquamation and erythema of the skin. However, in *TRPC7^+/−^* and *TRPC7^−/−^* mice, UVB exposure induced slight or no damage and less epidermal thickening than in wild‐type mice (Figure 3a–e). Furthermore, in wild‐type mice, UVB exposure increased the expression of epidermal differentiation markers KRT10 and KRT14, indicating the abnormal differentiation of keratinocytes; however, this effect was markedly attenuated in *TRPC7^+/−^* and *TRPC7^−/−^* mice (Figure 3f,g). Ca^2+^ is a known regulator of keratinocyte differentiation (Bikle, Xie, & Tu, 2012), and our results indicate that the abnormal differentiation induced by UVB was initiated by TRPC7‐mediated [Ca^2+^]_i_ elevation. After UVB exposure, we observed strong Ki‐67 staining in the cytoplasm but not in the nuclei of differentiating keratinocytes (Figure 3h), indicating that UVB may induce cell proliferation (El‐Abaseri, Putta, & Hansen, 2006) followed by abnormal differentiation. The shift from proliferation to differentiation may depend on the accumulation of oxidative DNA damage. Indeed, after UVB exposure, *TRPC7^+/−^* and *TRPC7^−/−^* mice showed less 8‐oxodG and TUNEL staining in the epidermis than did wild‐type mice, indicating reduced UVB‐induced oxidative DNA damage and fragmentation in these mice (Figure 3i,j). In addition, we observed the cytoplasmic expression of 8‐oxodG, which is associated with malignancy (Matosevic et al., 2015), in the dorsal skin of wild‐type mice but not *TRPC7^+/−^* or *TRPC7^−/−^* mice after UVB exposure (Figure 3i). SA‐β‐gal staining, used to analyze cell senescence, was also lower in the epidermis of *TRPC7^+/−^* and *TRPC7^−/−^* mice than in that of wild‐type mice after UVB exposure (Figure 3k). Together, these results provide evidence of a key role of TRPC7 channels in the physiologic and pathologic consequences of UVB irradiation.

### 2.4 TRPC7 mediates UVB‐induced epidermal aging in mice

**Figure 4:**
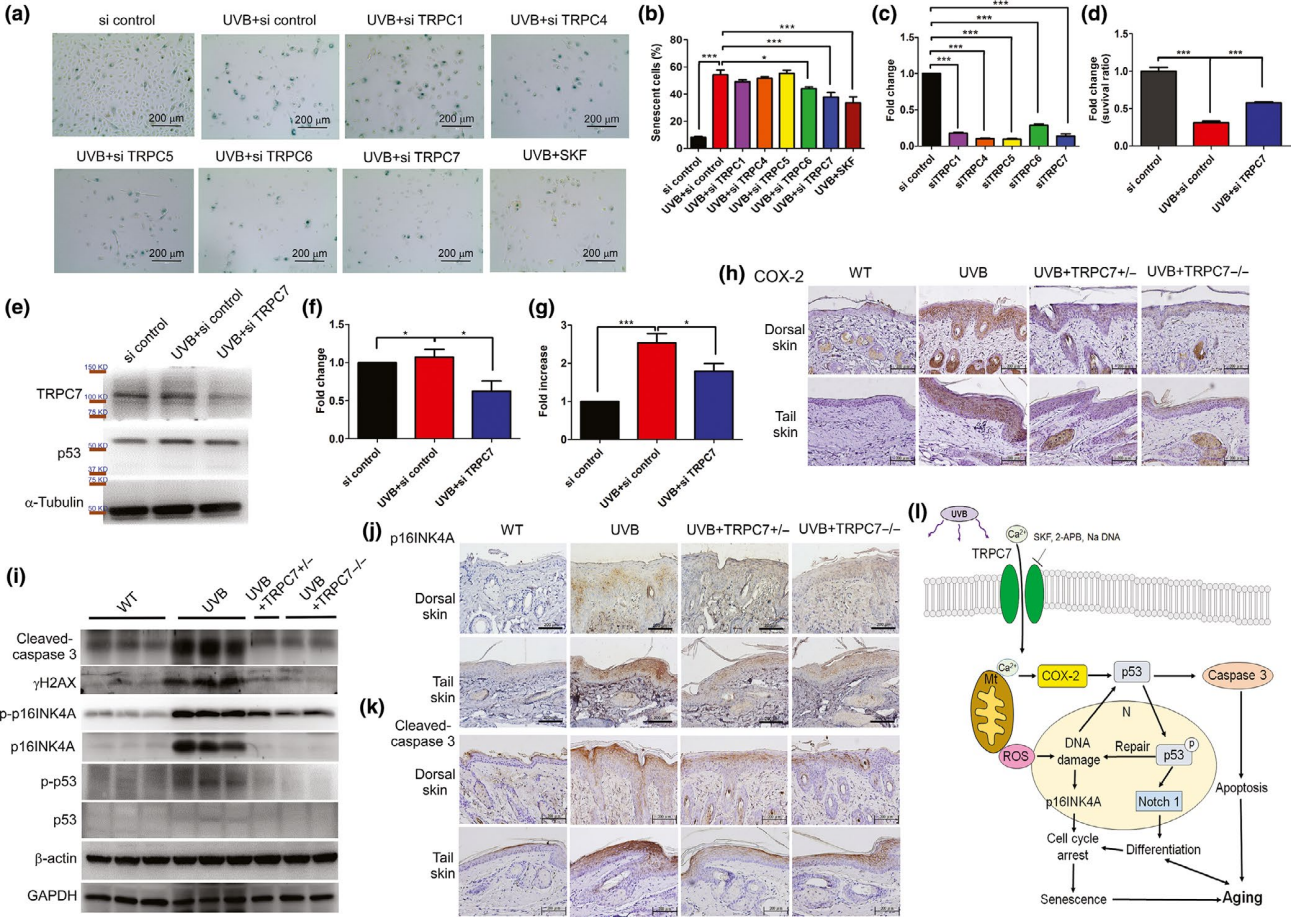
Ultraviolet B (UVB)‐induced pathology decreased in *TRPC7* knockdown cells. (a) After 24 hr of UVB exposure, senescent and nonsenescent cells were counted in various *TRPC* knockdown keratinocytes, and the ratio of the two was calculated (si = small interfering RNA). Cell senescence was determined by performing senescence‐associated β‐galactosidase (SA‐β‐gal) staining. (b) The percentage of senescent cells in >500 cells is shown for each group. (c) The efficiency of the knockdown of each *TRPC* siRNA is shown. (d) Knockdown of *TRPC7* increased the cell survival ratio after exposure to UVB. (e) Western blot analysis showing the expression of TRPC7, p53, and α‐tubulin in *TRPC7* knockdown cells. The quantification of (f) TRPC7 and (g) p53 protein expression in *TRPC7* knockdown cells is shown (data were normalized to the protein expression of the internal control, α‐tubulin). *p < .05; ***p < .001. (h) COX‐2 staining (brown, counterstained with hematoxylin) intensity was reduced in the skin of *TRPC7^−/−^* mice. (i) Western blot analysis showing the expression of cleaved caspase‐3, γH2AX, phospho‐p16INK4A (p‐p16INK4A), p16INK4A, phospho‐p53 (p‐p53), p53, β‐actin, and GAPDH. (j) The staining intensity of p16INK4A and (k) cleaved caspase‐3 (brown, counterstained with hematoxylin) was reduced in the skin of *TRPC7^−/−^* mice. (l) Schematic representation of how UVB‐induced epidermal aging is restrained by blocking *TRPC7*. UVB‐activated Ca^2+^ influx via TRPC7 initiates epidermal aging via an increase in Ca^2+^‐induced oxidative stress with p53 activation, oxidative DNA damage and fragmentation, cell senescence, abnormal differentiation, and apoptosis. This process is inhibited by TRPC inhibitors SKF, 2‐APB, and Na DNA. N, nucleus; Mt, mitochondria; ROS, reactive oxygen species

We found that after UVB irradiation, the knockdown of *TRPC7* in keratinocytes significantly decreased the percentage of senescent cells, as determined by SA‐β‐gal staining (Figure 4a–c), and improved the ratio of keratinocyte survival (Figure 4d) when compared with control cells. Furthermore, the knockdown of *TRPC7* inhibited UVB‐induced p53 expression in keratinocytes (Figure 4e–g), which was corroborated by our observation that the UVB‐induced activation of the p53 pathway was reduced in *TRPC7^+/−^* and *TRPC7^−/−^* mice (Figure 4i). In addition, extracellular Ca^2+^ influx is known to induce cyclooxygenase‐2 (COX‐2) expression, which increases p53 pathway activation and promotes the aging process (Hsu et al., 2015; Kim et al., 2016). We found that after UVB exposure, the high levels of COX‐2 protein expression observed in the epidermis of wild‐type mice were decreased to minimal levels in *TRPC7^+/−^* and *TRPC7^−/−^* mice (Figure 4h).

Although the DDR and cellular senescence are involved in the intermediate stages of the aging process, an imbalance of proliferation and apoptosis together with the accumulation of senescent and apoptotic cells results in cell aging (Cerella, Grandjenette, Dicato, & Diederich, 2016). We found that UVB exposure increased the levels of γH2AX, p16INK4A, and cleaved caspase‐3 in the epidermis of wild‐type mice, indicating increased senescence and apoptosis. Furthermore, the induction of γH2AX, p16INK4A, and cleaved caspase‐3 levels was attenuated in *TRPC7^+/−^* and *TRPC7^−/−^* mice (Figure 4i–k). These results confirm that UVB‐induced epidermal aging is attenuated in *TRPC7^+/−^* and *TRPC7^−/−^* mice, showing that TRPC7 is necessary for the initial increase in [Ca^2+^]_i_ and for activating the cascade of cellular processes that lead to skin aging (Figure 4l).

### 2.5 TRPC7 mediates UVB‐induced tumor initiation and growth

**Figure 5:**
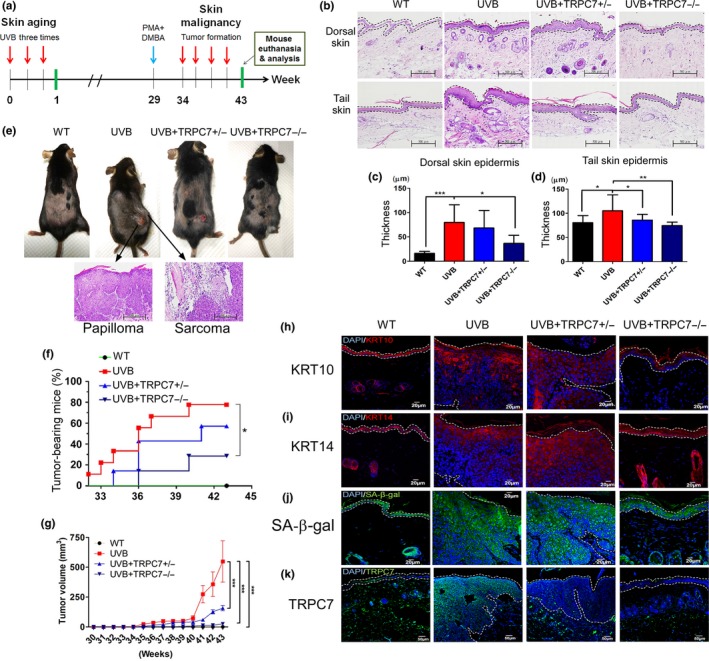
Cancer resistance in *TRPC7* knockout mice. (a) Experimental design for examining the effects of ultraviolet B (UVB) exposure and carcinogens on the epidermis of wild‐type (WT) and *TRPC7* knockout mice (*TRPC7^+/−^* and *TRPC7^−/−^*) for up to 43 weeks. UVB exposure was administered once every 2 days for 43 weeks. At week 29, the carcinogens phorbol myristate acetate (PMA, 7.5 μg) and 7,12‐dimethylbenz(a)anthracene (DMBA, 50 μg) were injected to promote tumor growth. (b) At week 43, dorsal skin and tail skin clippings were sectioned and stained with hematoxylin and eosin. The quantification of the mean (± *SD*) epidermal thickness in (c) dorsal and (d) tail skin. (e) Tumor formation, including epidermal papilloma and hypodermal sarcoma, was observed in the skin near the carcinogen injection sites. (f) Tumor prevalence and (g) tumor volume (mean ± standard error of the mean) increased with time in all groups of mice but were significantly reduced at 43 weeks in knockout mice compared with wild‐type mice. The expression of (h) KRT10, (i) KRT14, (j) senescence‐associated β‐galactosidase (SA‐β‐gal), and (k) TRPC7 is shown in mouse dorsal skin at the boundary between the epidermis and tumor. *p < .05; **p < .01; ***p < .001

Aging promotes hyperplastic pathologies, with cancer being one of the most serious (Campisi, 2013). We investigated whether interrupting UVB‐induced skin aging via the knockout of *TRPC7* reduced UVB‐induced skin tumorigenesis in mice. Aging‐associated tumorigenesis is dependent on the activation of the senescence‐associated secretory phenotype (SASP; Watanabe, Kawamoto, Ohtani, & Hara, 2017). We found that UVB‐induced SASP activation was reduced in the epidermis of *TRPC7^+/−^* and *TRPC7^−/−^* mice (Figure S3). In normal keratinocytes exposed to UVB, the p53 family of proteins is activated, and tissue repair is initiated (Campisi, 2013). Despite repeated UVB irradiation for 10 weeks and epidermal thickening at 1 week, the epidermis in all UVB‐exposed mice was repaired and restored to a single layer at the 10th week, resembling nonirradiated skin (Figure S4). Once the repair system no longer provides sufficient recovery from injury, however, the aging process begins to advance in the damaged tissue, and the epidermis thickens. In *TRPC7^+/−^* and *TRPC7^−/−^* mice, we observed the attenuation of this pathology (Figure 5a–d). At week 29, UVB‐irradiated mice were injected with carcinogens, and the prevalence and volume of papilloma and sarcoma tumor began to increase by week 34 in wild‐type mice (Figure 5e). Significantly fewer *TRPC7^+/−^* and *TRPC7^−/−^* mice than wild‐type mice developed tumors (Figure 5f), and the tumors that did develop in *TRPC7^+/−^* and *TRPC7^−/−^* mice were of significantly smaller volume (Figure 5g), although *TRPC7* deficiency did not completely eliminate tumor formation. In addition, levels of the differentiation markers KRT10 and KRT14 and senescence marker SA‐β‐gal were upregulated at the boundary of the tumor in UVB‐exposed wild‐type mice (Figure 5h–j), suggesting that SASP activation and carcinogen‐stimulated tumor formation resulted from cell senescence. We also observed the overexpression of TRPC7 in UVB‐exposed skin, especially in papilloma (Figure 5k). Our analysis of the pathologic features of cancer in human tissues also supported the hypothesis that TRPC7 promotes cancer progression by mediating tumor growth (Table S2). Furthermore, TRPC7 was found to be overexpressed in tumor biopsies from patients with non‐small cell lung cancer (Figure S5). The overexpression of TRPC7 that we observed in tumors from mouse and humans raises the possibility that TRPC7 promotes tumorigenesis.

### 2.6 UVB‐induced *p53* mutations are prevented in *TRPC7* knockout mice

**Figure 6:**
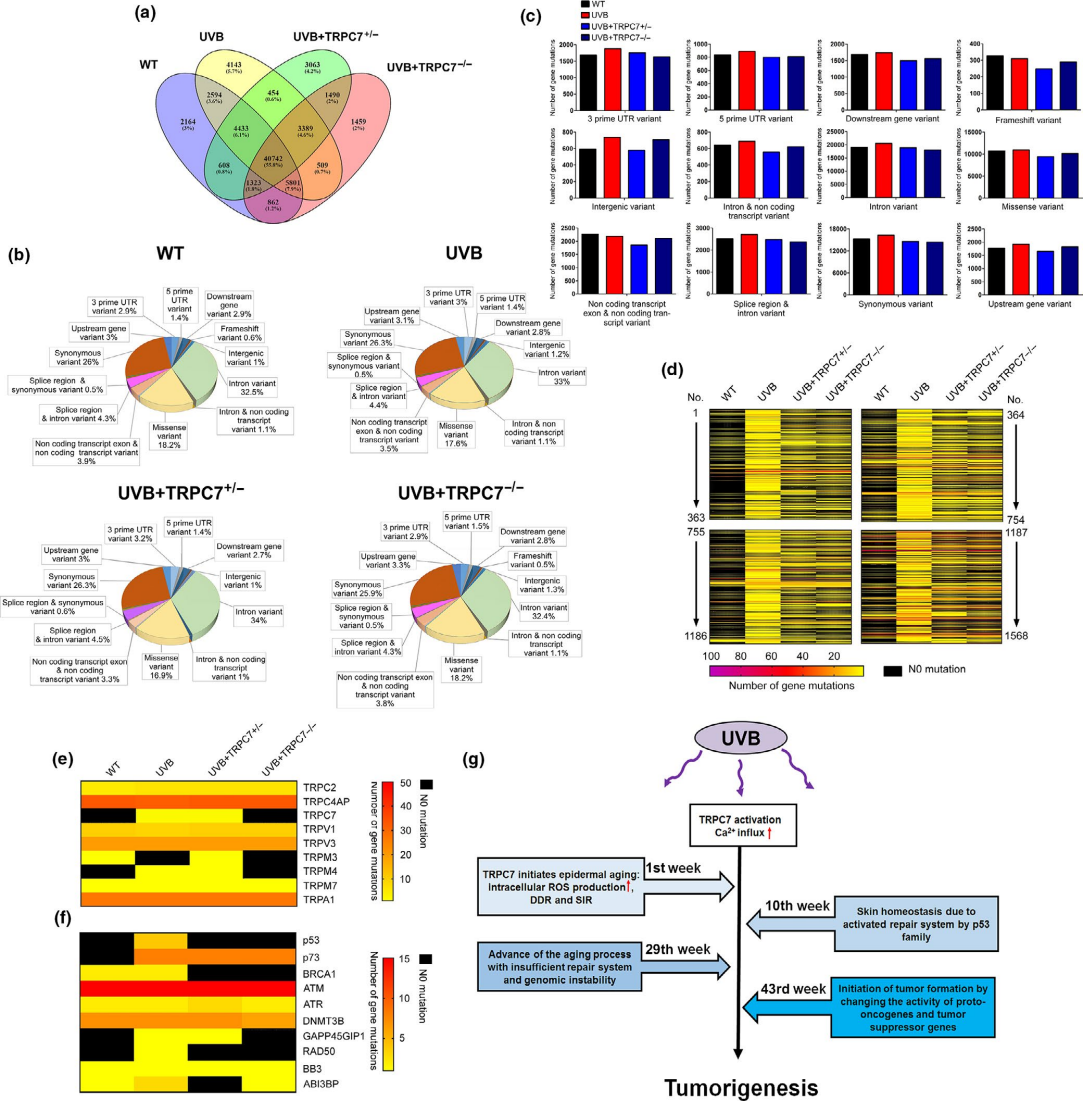
*TRPC7* knockout reduced the number of gene mutations in ultraviolet B (UVB)‐induced tumorigenesis. Whole‐exome sequencing was used to analyze the number of mutations in the dorsal skin of UVB‐exposed wild‐type (WT) and *TRPC7* knockout mice (*n* = 3). Merged data from whole‐exome sequencing indicated (a) the number of mutations, (b) the contribution of each variant type, and (c) the number of mutations of each variant type. (d) A heat map illustrates the number of mutations from UVB‐induced tumorigenesis, (e) the mutations specific to the TRP gene family, and (f) the p53 family genes and p53‐dependent molecules. (g) Schematic representation of the role of TRPC7 as a potential tumor initiator gene in ultraviolet B (UVB)‐induced aging and cancer progression. UVB‐activated TRPC7 initiates UVB‐induced skin aging via intracellular Ca^2+^ elevation, resulting in oxidative stress, DNA damage response (DDR) activation, abnormal differentiation, and senescence inflammation response (SIR) activation; this pathology is repaired with the activation of p53 to maintain tissue homeostasis. When homeostasis is no longer maintained and the aging process is activated, carcinogens promote cancer progression. TRPC7 initiates tumorigenesis by causing genomic instability, thereby changing the activity of proto‐oncogenes and tumor suppressor genes. TRPC7 is also overexpressed in cancer progression, which may promote the cell cycle and cytoskeletal remodeling by increasing Ca^2+^ signaling

To examine how TRPC7 affects UVB‐induced tumorigenesis, we performed whole‐exome sequencing in the dorsal skin of mice to characterize genetic mutations. We found that *TRPC7^+/−^* and *TRPC7^−/−^* mice had the smallest percentage of mutations, despite being exposed to UVB (Figure 6a). Further analysis did not reveal significant differences between groups (Figure 6b), but UVB‐exposed *TRPC7^+/−^* and *TRPC7^−/−^* mice consistently had fewer mutations of each variant type than did UVB‐exposed wild‐type mice (Figure 6c). Whole‐exome sequencing also showed that *TRPC7^+/−^* and *TRPC7^−/−^* mice had a lower number of mutations than did wild‐type mice (Figure 6d). Interestingly, two TRP channel genes (*TRPC7* and *TRPM4*) that were presumably mutated from UVB‐induced tumorigenesis exhibited a greater number of mutations in wild‐type mice than in *TRPC7^+/−^* or *TRPC7^−/−^* mice (Figure 6e). UVB exposure in wild‐type mice also induced mutations in p53 family genes and in p53‐dependent genes (Figure 6f); in contrast, *TRPC7^+/−^* and *TRPC7^−/−^* mice had no mutations in p53 and fewer mutations in the genes associated with DNA repair, such as *ATR*, *DNMT3B*, and *RAD50* (Figure 6f). These results showing that p53 and p53‐dependent DNA repair molecules were mutated less frequently after UVB exposure and thus maintained their protective functions against UVB‐induced tumorigenesis in *TRPC7* knockout mice support our findings that tumor formation was reduced in *TRPC7* knockout mice (Figure 5). Together, our data suggest that, in response to UVB, TRPC7 is a primary initiator of epidermal aging and skin tumorigenesis, contributes to mutations in the *p53* gene family, and promotes the development of cancerous tumors, indicating a novel role of nociceptive TRPC7 in mediating the aging process and cancer progression.

## 3 DISCUSSION

In this study, we show evidence for a role of *TRPC7* as a potential tumor initiator gene in UVB‐induced epidermal aging and skin tumorigenesis. After UVB irradiation, the increased level of diacylglycerol in the cell membrane immediately activates TRPC7 (Hsu et al., 2015), and increased Ca^2+^ influx from TRPC7 in turn further upregulates TRPC7 levels (Figure 4e). Several Ca^2+^‐dependent transcription factors, such as nuclear factor (NF)‐κB, cAMP response element binding protein, and nuclear factor of activated T cells protein may be involved in controlling TRPC7 upregulation to amplify its activation. Nonetheless, our findings indicate that TRPC7 induces Ca^2+^ influx to produce a snowball effect that triggers several signaling pathways to initiate epidermal ROS production, inflammation, DNA damage, senescence, and cell aging. The function of *TRPC7* as a potential tumor initiator gene is supported by our findings that it initiates tumor progression by causing cell aging and genomic instability, followed by a change in the activity of proto‐oncogenes and tumor suppressor genes to promote tumorigenesis. We show for the first time to our knowledge that TRPC7 is a nociceptive mechanoreceptor that actively participates in all stages of tumorigenesis. This provides a mechanistic link between TRP channels and their putative roles in cancer metastasis by altering cellular Ca^2+^ homeostasis (Fels, Bulk, Petho, & Schwab, 2018).

According to the theory of tumorigenesis, the DDR triggers the senescence inflammation response (SIR) and SASP activation, and genomic instability results in the activation of oncogenes and the dysfunction of tumor suppressor genes (Pribluda et al., 2013). SIR‐induced *p53* gene family activation drives the DNA repair system to recover from genomic instability and maintain tissue homeostasis; however, the dysfunction of *p53* family genes (or tumor suppressor genes) that results from genomic instability can promote tumorigenesis (Eischen, 2016). Treatment with nonsteroidal anti‐inflammatory drugs as tumor preventive agents restrains tumorigenesis by blocking inflammation (Gurpinar, Grizzle, & Piazza, 2014). Although the activation of p53 family genes drives tissue homeostasis, the repeated oncogenic stress from UVB irradiation breaks the balance between genomic instability and the DNA repair system (Pribluda et al., 2013), and Ca^2+^ signals from UVB‐activated TRPC7 initiate tumorigenesis through genomic instability. Furthermore, senescence stimulates tumor formation via SASP activation (Figure 6g). Tumorigenesis, however, is a complex process, and several other tumor initiator genes may be involved in regulating UVB‐induced tumor development. Although the blockage of TRPC7 inhibits tumor initiation, it cannot completely prohibit tumor formation.

Our discovery that TRPC7 is a nociceptive mechanoreceptor, together with our previous finding that TRPC7 has a role in UVB‐induced skin aging via increased ROS production (Hsu et al., 2015), is consistent with the previously reported links between nociceptive TRP receptors and aging (Buffenstein, 2008; Park et al., 2008; Riera et al., 2014; Seluanov et al., 2009). Interestingly, TRP channel expression has been previously shown to increase with age‐related degeneration (Santoni & Farfariello, 2011; Takada et al., 2013; Yue et al., 2015), but our results suggest that TRP channels, especially nociceptive TRP channels, are also important in initiating the aging process. Nociceptive TRP channels respond to environmental stimuli, such as pain, cold, or heat (Venkatachalam & Montell, 2007). The process of aging and age‐associated diseases may be due to excess Ca^2+^ signaling from active nociceptors upon continual environmental stimuli. Interestingly, we found that 55.8% of gene mutations occurred through the natural process of skin aging, but only UVB treatment facilitated the induction of tumorigenesis (Figure 6a). This supports that mutations occur during the natural process of skin aging but that an external trigger such as UVB is required for aging‐associated diseases. According to our study, environmental stimulus with UVB activates TRPC7, which is pivotal to the initiation of tumorigenesis associated with skin aging. The possibility remains that aging and aging‐associated diseases are caused by the same source of active nociceptors. Blockage of nociceptor activation represents a new strategy for maintaining healthy longevity.

Increased Ca^2+^ influx may result in genomic instability; however, how alternative Ca^2+^ homeostasis directly induces tumorigenesis remains unclear. Although cellular senescence depends on the arrest of cell proliferation, we speculate that Ca^2+^ influx via TRPC7 simultaneously activates Ca^2+^‐activated K^+^ channels to reduce the intracellular K^+^ concentration, which activates cell proliferation (Kunzelmann, 2005; Lehen'kyi, Shapovalov, Skryma, & Prevarskaya, 2011). Thus, TRP channel upregulation potentially maintains cell proliferation, giving way to tumorigenesis. TRPC7 channels may maintain cell proliferation to a greater extent than previously believed because they have a large permeability ratio [p(Ca^2+^/Na^+^) = 0.7–5.0] and sufficient conductance (25–50 pS; Gees, Colsoul, & Nilius, 2010; Owsianik, Talavera, Voets, & Nilius, 2006). Thus, the activation of TRPC7 may have a greater, more immediate effect on Ca^2+^ entry than does the activation of other TRP channels. If Ca^2+^ entry from TRPC7 is important to maintain tumorigenesis, malignant cells potentiate intensive epigenetic regulation and mutation according to the evolution of a tumor (Coyle, Boudreau, & Marcato, 2017). In our study, we found that TRPC7 upregulation in tumors promotes tumor growth and also maintains genomic instability. Additionally, in *TRPC7* knockout mice, p53 was protected from mutations because of the disruption of cell senescence. These results suggest that the expression of mutated *p53* may be mediated through epigenetic regulation by UVB‐activated TRPC7. Epigenetic reactivation of tumor suppressor genes can be determined by Ca^2+^ signaling, and targeting epigenetic pathways is a potential approach for cancer therapy (Raynal et al., 2016). Therefore, it is conceivable that, after the UVB‐induced mutation of *p53*, the expression of mutated *p53* is suppressed epigenetically, and the TRPC7‐mediated epigenetic reactivation of mutated *p53* in turn promotes tumorigenesis.

In conclusion, we examined the mechanism underlying cancer resistance and longevity resulting from reduced nociceptive TRP channel expression (Riera et al., 2014). Although nociceptive TRP channels transduce stimulation into neuronal impulses for perception, they are also “first responders,” with an influx of Ca^2+^ to protect tissue against harm from extreme mechanical pressure, temperature, or irradiation. Consistent with this first responder role of TRP channels, our results show that harmful UVB exposure induces Ca^2+^ influx through nociceptive TRPC7 channels. TRPC7 activation, however, also initiates skin aging, results in mutations in the p53 gene family, and promotes the development of cancerous tumors. Indeed, our findings suggest that TRPC7 is a potential tumor initiator gene in tumorigenesis. Finally, our results may explain why the blocking or lack of nociceptive TRP channels facilitates pain relief, cancer resistance, and longevity.

## 4 MATERIALS AND METHODS

### 4.1 Chemicals

Thapsigargin (TG), 2‐aminoethoxydiphenyl borate (2‐APB), H_2_O_2_, icilin, ATP, 1‐oleoyl‐2‐acetyl‐sn‐glycerol (OAG), SKF96365, LaCl_3_, ethylenediaminetetraacetic acid (EDTA), dihydroethidium (DHE), phorbol myristate acetate (PMA), 7,12‐dimethylbenzathracene (DMBA), and all other reagents were purchased from Sigma‐Aldrich. Fluo‐4‐AM and caged IP_3_ were purchased from Thermo Fisher Scientific.

### 4.2 Cell culture

Human primary keratinocytes were purchased from Lonza. Keratinocytes were incubated at 37°C in humidified 5% CO_2_ in serum‐free keratinocyte growth medium supplemented with human recombinant epidermal growth factor, bovine pituitary extract (5 μg/ml each), human insulin‐like growth factor I (1 μg/ml), and hydrocortisone (5 μg/ml; Gibco BRL).

### 4.3 Calcium imaging

To examine which TRP channels were involved in UVB‐induced Ca^2+^ elevation**,** intracellular Ca^2+^ responses in human keratinocytes were induced by pretreatment with 20 nM TG, 2 μM 2‐APB, 100 μM H_2_O_2_, 100 μM icilin, 200 μM ATP, or 100 μM OAG combined with each TRP channel's inhibitor before UVB irradiation, as previously described (Hsu et al., 2015). Before the experiments, cells were loaded with 1 μM Fluo‐4‐AM (Molecular Probes) at 37°C for 20 min and then washed with balanced salt solution buffer (5.4 mM KCl, 5.5 mM d‐glucose, 1 mM MgSO_4_, 130 mM NaCl, 20 mM HEPES [pH 7.4], and 2 mM CaCl_2_). Intracellular Ca^2+^ concentrations ([Ca^2+^]_i_) were calculated from the ratio of fluorescence intensities (in the absence of Ca^2+^ and at saturation) that were emitted at 509 nm upon excitation with consecutive 3‐s pulses of 488‐nm light at a resolution of 1,376 × 1,038 pixels by using an Olympus Cell^R IX81 fluorescence microscope (Olympus) equipped with an MT 20 illumination system (Olympus) and UPLanApo 10× objective lens. [Ca^2+^]_i_ was estimated based on a [Ca^2+^] calibration curve created by using a Ca^2+^ Calibration Buffer Kit (Thermo Fisher Scientific). [Ca^2+^]_i_ was calculated by using Fluo‐4 excited at 488 nm and imaged at 20°C by using the same instrument. Fluo‐4 signals were calibrated by measuring the fluorescence intensity from microcuvettes containing 10 mM K_2_‐EGTA (pH 7.20) buffered to various Ca^2+^ concentrations. [Ca^2+^]_i_ was calculated by using the following formula: [Ca^2+^]_free_ = *K*_d_ *(*F* − *F*_min_/*F*_max_ − *F*). Plotting the fluorescence intensity versus [Ca^2+^]_i_ yielded the calibration curve with the formula of: [Ca^2+^]_i_ = *K*_d_ *(*F* − *F*_min_/*F*_max_ − *F*), where *K*_d_ = 345 nM, *F* = Fluo‐4 intensity, *F*_max_ = 640, and *F*_min_ = 21.7 for Fluo‐4.

### 4.4 Focal IP_3_ uncaging

Caged IP_3_ was uncaged by using photolysis with UV light (300–400 nm) to investigate the activation of TRPC1, TRPC4, and TRPC5. For photolytic uncaging, an Olympus FV1000 MPE multiphoton laser scanning microscope equipped with an argon laser was used to produce a collimated light beam as the principal uncaging laser line at λ = 408 nm. To detect the effect of IP_3_ on TRPC Ca^2+^ responses, keratinocytes were plated on 2.4‐mm coverslips in a 4‐cm dish. After 50 mg/cm^2^ UVB irradiation, cells were pretreated with 2 μM caged IP_3_ for 30 min and stained with the Ca^2+^ dye Fluo‐4 to analyze changes in intracellular Ca^2+^ levels. Ca^2+^‐induced fluorescence was observed using an Olympus FV1000 laser scanning microscope. Caged IP_3_ was uncaged via illumination with UV light (λ = 408 nm), and IP_3_ molecule released was immediately available to bind IP_3_ receptors and TRPCs.

### 4.5 Intracellular ROS measurement

Cells were stained with 5 μM dihydroethidium (free radical indicator), and the intensity of emitted fluorescence was analyzed by using an Olympus FV1000 laser scanning microscope.

### 4.6 Quantitative reverse transcription–polymerase chain reaction (qRT‐PCR)

After transfection with each siRNA (control siRNA, si*TRPC1*, si*TRPC4*, si*TRPC5*, si*TRPC6*, and si*TRPC7*) for 24 hr, total RNA was extracted from keratinocytes by using TRIzol reagent (Thermo Fisher Scientific). Complementary DNA was synthesized from RNA (1 μg) by using a reverse transcriptase kit (Thermo Fisher Scientific). Incubation conditions included 10 min at 25°C, 120 min at 37°C, and 5 min at 85°C. The resulting complementary DNAs were used to detect the expression of each *TRPC* gene in skin (*TRPC1*, *TRPC4*, *TRPC5*, *TRPC6*, and *TRPC7*) by performing quantitative PCR with a TaqMan Gene Expression Assay kit (Thermo Fisher Scientific).

### 4.7 TRPC7 knockout mice and UVB treatment protocol

*TRPC7* knockout mice were purchased as heterozygous *TRPC7 ^+/- ^*mice (TRPC7^tm1.1Lbi^/Mmjax) from Mutant Mouse Resource and Research Center (MMRRC). Our *TRPC7^+/+^*, *TRPC7^+/−^*, and *TRPC7^−/−^* mouse colonies were housed at the National Laboratory Animal Center (NLAC), and animal experiments were performed at Kaohsiung Medical University in Taiwan. To examine skin pathology after UVB exposure, 8‐week‐old mice were exposed to 360 mJ/cm^2^ UVB (radiation 280–320 nm) three times weekly, and skin aging and tumor development were monitored. All animal experiments were performed under an affidavit of approval of animal use protocol at Kaohsiung Medical University (IACUC approval number 103122).

### 4.8 Immunohistochemistry staining of skin tissue

Dorsal and tail skin from *TRPC7^+/+^*, *TRPC7^+/−^*, and *TRPC7^−/−^* mice were fixed and embedded in paraffin. Antibodies against Ki‐67 (proliferation marker; Abcam), COX‐2 (cyclooxygenase‐2; Abcam), P16INK4A (senescence marker; Sigma‐Aldrich), caspase‐3 (activated form, apoptosis mediator; GeneTex), interleukin‐6 (IL‐6; GeneTex), tumor necrosis factor alpha (TNF‐α; GeneTex), or regulated upon activation normal T cell express sequence (RANTES, also known as CCL5; GeneTex) were used to detect target molecules. Immunoreactivity was visualized after incubation with 3,3′diaminobenzidine (DAB) substrate‐chromogen solution (DAKO) according to the manufacturer's protocol.

### 4.9 Immunofluorescence staining

Fixed keratinocytes or paraffin‐embedded skin sections were incubated with primary antibodies against TRPC7 (GeneTex), KRT10 (keratin 10; Proteintech), KRT14 (keratin 14; Proteintech), 8‐oxodG (8‐hydroxy‐2′‐deoxyguanosine; Abcam), or SA‐β‐gal (senescence‐associated β‐galactosidase; Abcam) at 4°C overnight. Cells or skin sections were subsequently incubated with secondary antibody for 1 hr and then with 4′,6‐diamidino‐2‐phenylindole (DAPI, Thermo Fisher Scientific) for 5 min. Tissue was mounted on coverslips that were then inverted and fixed onto glass slides by using antifade mounting reagent (Biotium). Fluorescence imaging was performed by using a confocal microscope (Olympus FV1000).

### 4.10 Western blot analysis

Western blot analysis was performed with whole‐cell lysates. Briefly, cells were lysed by incubation on ice for 30 min in M‐PER Mammalian Protein Extraction Reagent (Thermo Fisher Scientific) containing proteinase and phosphatase inhibitors. Cell debris were removed by centrifugation at 10,000 *g* for 10 min at 4°C. The protein concentration of cell lysates was determined by using the Bradford method (Bio‐Rad). Proteins (50 μg) in cell lysates were resolved by performing sodium dodecyl sulfate–polyacrylamide gel electrophoresis in a 10% gel and then transferred to a Hybond‐P polyvinylidene fluoride membrane (Amersham Biosciences). The membrane was first incubated with primary antibodies against TRPC7 (GeneTex), p53 (Cell Signaling Technology), phospho‐p53 (Abcam), γH2AX (a marker of DNA double‐strand breaks; Novus Biologicals), P16INK4A (Sigma‐Aldrich), phospho‐P16INK4A (Sigma‐Aldrich), caspase‐3 (activated form; GeneTex), interleukin‐1β (IL‐1β; GeneTex), IL‐6 (GeneTex), TNF‐α (GeneTex), gamma interferon‐induced protein (IP‐10, also known as CXCL10; Novus Biologicals), RANTES (GeneTex), plasminogen activator inhibitor‐1 (PAI‐1; GeneTex), β‐actin (Sigma‐Aldrich), α‐tubulin (Santa Cruz Biotechnology), or GAPDH (GeneTex) and then with horseradish peroxidase‐conjugated secondary antibodies. Immunoreactive proteins were visualized by using enhanced chemiluminescence reagents (Amersham Biosciences).

### 4.11 TRPC knockdown in human keratinocytes

For *TRPC* knockdown experiments, human primary keratinocytes were transfected with si*TRPC1*, siTRPC4, si*TRPC5*, si*TRPC6*, si*TRPC7*, or sicontrol (Santa Cruz) constructs by using GenMute™ siRNA Transfection Reagent (SignaGen Laboratories). After transfection, healthy, subconfluent keratinocytes were cultured for 48 hr before undergoing further analysis. The expression of target genes was validated by using qRT‐PCR or Western blot analysis.

### 4.12 Mechanical sensitivity

Mechanical sensitivity was analyzed by examining withdrawal thresholds to a series of von Frey filaments (each calibrated to exert 0.008, 0.02, 0.04, 0.07, 0.16, 0.4, 0.6, 1, or 2 g of force) applied to the paw. The smallest force that produced a withdrawal was recorded as the withdrawal threshold. von Frey filaments were applied 10 times to the paw, once every second. Researchers were blinded to the animal's status during evaluation.

### 4.13 Ethics committee approval

Non‐small cell lung cancer samples from patients with adenocarcinoma and squamous cell carcinoma were collected in accordance with the relevant guidelines and regulations approved by the Institutional Review Board/Ethics Committee (IRB) at Chi Mei Medical Center, Liouying, Tainan, Taiwan (serial no.: 10405‐L01). These patient samples were used in research entitled “Role of TRPC7 in cancer progression study.” Informed consent was obtained from each participant. A total of 100 non‐small cell lung cancer samples were collected between May 2016 and June 2017 and were further analyzed by using immunohistochemistry.

### 4.14 Whole‐exome sequencing

Genomic DNA was isolated from the dorsal skin of mice from each of the four groups by using Qiagen DNeasy Blood and Tissue Kit (Qiagen). The quality of the isolated DNA was examined by using an Agilent 2100 Bioanalyzer System according to the manufacturer's protocols (Agilent Technologies). DNA that passed quality control was sheared into fragments ranging from 150 to 200 bp. Ends were repaired by using blunt‐ended fragments with 5′‐phosphorylated ends. Specific adaptors obtained from Illumina were ligated to the fragments by using adaptor‐modified ends. The unligated adapters were removed by using AMPure XP beads, and the purified samples were amplified by using PCR with SureSelect Primer and SureSelect Pre‐capture Reverse PCR primers to generate the library for further analysis. After hybridization was performed by using the exome capture library, samples were amplified with indexed tagging primers by using PCR. The quality and quantity of the enriched library were examined by using an Agilent 2100 Bioanalyzer System and real‐time PCR. The samples were then sequenced by using an Illumina Hiseq 4000 (Illumina) according to the manufacturer's standard protocols. A total of five gigabases of DNA base‐pair sequences were read, and the coverage of the whole‐exome sequencing was approximately 100‐fold.

### 4.15 Statistical analysis

GraphPad Prism was used to generate bar charts; error bars indicated standard deviations unless otherwise noted. Analysis of variance and Student's *t* tests were used to compare the differences between groups. In addition, the differences between *TRPC7* expression levels, clinical pathology features, and tumor prevalence were compared by using chi‐squared tests. A *p*‐value of less than .05 for the difference between groups was considered statistically significant.

## CONFLICT OF INTEREST

J.S.K. is owner of BPS International, which has received consulting fees from Agendia Incorporated, OncoSec Medical Incorporated, and Veridex, LLC, a Johnson & Johnson company (not related to this work). All other authors declare no competing interests.

## AUTHOR CONTRIBUTIONS

W.L.H., M.H.T., C.H.C., and T.Y. designed the experiments. C.J.Y., W.L.H., Y.C.H., Y.Y.H., L.C.L., T.F.T., and M.H.T. coordinated and performed all animal experiments and tissue analyses. C.Y.W. and J.L.L. collected samples from patients and assisted with clinical data analysis. W.L.H., J.H.L., and H.S.Y. cultured primary keratinocytes and performed the cellular imaging study of Ca^2+^ dynamics and intracellular reactive oxygen species detection. M.H.T. performed the statistical analysis. C.T.Y. and H.S.Y. provided advice on the project. W.L.H., M.H.T, J.S.K., C.H.C., and T.Y. wrote the manuscript. All authors reviewed and approved the final manuscript.

## References

[acel13075-bib-0001] Bikle, D. D. , Xie, Z. , & Tu, C. L. (2012). Calcium regulation of keratinocyte differentiation. Expert Review of Endocrinology & Metabolism, 7, 461–472. 10.1586/eem.12.34 23144648 PMC3491811

[acel13075-bib-0002] Buffenstein, R. (2008). Negligible senescence in the longest living rodent, the naked mole‐rat: Insights from a successfully aging species. Journal of Comparative Physiology B, 178, 439–445. 10.1007/s00360-007-0237-5 18180931

[acel13075-bib-0003] Campisi, J. (2013). Aging, cellular senescence, and cancer. Annual Review of Physiology, 75, 685–705. 10.1146/annurev-physiol-030212-183653 PMC4166529 23140366

[acel13075-bib-0004] Cerella, C. , Grandjenette, C. , Dicato, M. , & Diederich, M. (2016). Roles of apoptosis and cellular senescence in cancer and aging. Current Drug Targets, 17, 405–415. 10.2174/1389450116666150202155915 25642721

[acel13075-bib-0005] Coyle, K. M. , Boudreau, J. E. , & Marcato, P. (2017). Genetic mutations and epigenetic modifications: Driving cancer and informing precision medicine. BioMed Research International, 2017, 9620870 10.1155/2017/9620870 28685150 PMC5480027

[acel13075-bib-1006] Eischen, C. M. (2016). Genome Stability Requires p53. Cold Spring Harb Perspect Med, 6, a026096 http://doi:10.1101/cshperspect.a026096 27252396 10.1101/cshperspect.a026096 PMC4888814

[acel13075-bib-0006] El‐Abaseri, T. B. , Putta, S. , & Hansen, L. A. (2006). Ultraviolet irradiation induces keratinocyte proliferation and epidermal hyperplasia through the activation of the epidermal growth factor receptor. Carcinogenesis, 27, 225–231. 10.1093/carcin/bgi220 16123117

[acel13075-bib-0007] Fels, B. , Bulk, E. , Petho, Z. , & Schwab, A. (2018). The role of TRP channels in the metastatic cascade. Pharmaceuticals, 11, 48 10.3390/ph11020048 PMC6027473 29772843

[acel13075-bib-0008] Gees, M. , Colsoul, B. , & Nilius, B. (2010). The role of transient receptor potential cation channels in Ca2+ signaling. Cold Spring Harbor Perspectives in Biology, 2, a003962 10.1101/cshperspect.a003962 20861159 PMC2944357

[acel13075-bib-0009] Gurpinar, E. , Grizzle, W. E. , & Piazza, G. A. (2014). NSAIDs inhibit tumorigenesis, but how? Clinical Cancer Research, 20, 1104–1113. 24311630 10.1158/1078-0432.CCR-13-1573 PMC3947450

[acel13075-bib-0010] Harteneck, C. , & Leuner, K. (2014).TRP channels in neuronal and glial signal transduction In Heinbockel T. (Ed.), Neurochemistry. London: IntechOpen.

[acel13075-bib-0011] Hsu, W. L. , Lu, J. H. , Noda, M. , Wu, C. Y. , Liu, J. D. , Sakakibara, M. , … Yoshioka, T. (2015). Derinat protects skin against ultraviolet‐B (UVB)‐induced cellular damage. Molecules, 20, 20297–20311. 10.3390/molecules201119693 26569211 PMC6331914

[acel13075-bib-0012] Kim, J. , Vaish, V. , Feng, M. , Field, K. , Chatzistamou, I. , & Shim, M. (2016). Transgenic expression of cyclooxygenase‐2 (COX2) causes premature aging phenotypes in mice. Aging, 8, 2392–2406. 10.18632/aging.101060 27750221 PMC5115895

[acel13075-bib-0013] Kunzelmann, K. (2005). Ion channels and cancer. Journal of Membrane Biology, 205, 159–173. 10.1007/s00232-005-0781-4 16362504

[acel13075-bib-0014] Lehen'kyi, V. , Shapovalov, G. , Skryma, R. , & Prevarskaya, N. (2011). Ion channels and transporters in cancer. 5. Ion channels in control of cancer and cell apoptosis. American Journal of Physiology: Cell Physiology, 301, C1281–C1289. 21940667 10.1152/ajpcell.00249.2011

[acel13075-bib-0015] Masaki, H. , Izutsu, Y. , Yahagi, S. , & Okano, Y. (2009). Reactive oxygen species in HaCaT keratinocytes after UVB irradiation are triggered by intracellular Ca(2+) levels. Journal of Investigative Dermatology. Symposium Proceedings, 14, 50–52. 10.1038/jidsymp.2009.12 19675553

[acel13075-bib-0016] Matosevic, P. , Klepac‐Pulanic, T. , Kinda, E. , Augustin, G. , Brcic, I. , & Jakic‐Razumovic, J. (2015). Immunohistochemical expression of 8‐oxo‐7,8‐dihydro‐2′‐deoxyguanosine in cytoplasm of tumour and adjacent normal mucosa cells in patients with colorectal cancer. World Journal of Surgical Oncology, 13, 241 10.1186/s12957-015-0667-6 26245656 PMC4527254

[acel13075-bib-0017] Omerbasic, D. , Smith, E. S. , Moroni, M. , Homfeld, J. , Eigenbrod, O. , Bennett, N. C. , … Lewin, G. R. (2016). Hypofunctional TrkA accounts for the absence of pain sensitization in the African naked mole‐rat. Cell Reports, 17, 748–758. 10.1016/j.celrep.2016.09.035 27732851 PMC5081396

[acel13075-bib-0018] Owsianik, G. , Talavera, K. , Voets, T. , & Nilius, B. (2006). Permeation and selectivity of TRP channels. Annual Review of Physiology, 68, 685–717. 10.1146/annurev.physiol.68.040204.101406 16460288

[acel13075-bib-0019] Park, T. J. , Lu, Y. , Juttner, R. , Smith, E. S. , Hu, J. , Brand, A. , … Lewin, G. R. (2008). Selective inflammatory pain insensitivity in the African naked mole‐rat (Heterocephalus glaber). PLoS Biology, 6, e13 10.1371/journal.pbio.0060013 18232734 PMC2214810

[acel13075-bib-0020] Park, T. J. , Reznick, J. , Peterson, B. L. , Blass, G. , Omerbasic, D. , Bennett, N. C. , … Lewin, G. R. (2017). Fructose‐driven glycolysis supports anoxia resistance in the naked mole‐rat. Science, 356, 307–311. 10.1126/science.aab3896 28428423

[acel13075-bib-0021] Phelan, K. D. , Shwe, U. T. , Abramowitz, J. , Birnbaumer, L. , & Zheng, F. (2014). Critical role of canonical transient receptor potential channel 7 in initiation of seizures. Proceedings of the National Academy of Sciences of the United States of America, 111, 11533–11538. 10.1073/pnas.1411442111 25049394 PMC4128138

[acel13075-bib-0022] Pribluda, A. , Elyada, E. , Wiener, Z. , Hamza, H. , Goldstein, R. E. , Biton, M. , … Ben‐Neriah, Y. (2013). A senescence‐inflammatory switch from cancer‐inhibitory to cancer‐promoting mechanism. Cancer Cell, 24, 242–256. 10.1016/j.ccr.2013.06.005 23890787

[acel13075-bib-0023] Raynal, N. J. , Lee, J. T. , Wang, Y. , Beaudry, A. , Madireddi, P. , Garriga, J. , … Issa, J. P. (2016). Targeting calcium signaling induces epigenetic reactivation of tumor suppressor genes in cancer. Cancer Research, 76, 1494–1505. 10.1158/0008-5472.CAN-14-2391 26719529 PMC4794357

[acel13075-bib-0024] Riera, C. E. , Huising, M. O. , Follett, P. , Leblanc, M. , Halloran, J. , Van Andel, R. , … Dillin, A. (2014). TRPV1 pain receptors regulate longevity and metabolism by neuropeptide signaling. Cell, 157, 1023–1036. 10.1016/j.cell.2014.03.051 24855942

[acel13075-bib-0025] Santoni, G. , & Farfariello, V. (2011). TRP channels and cancer: New targets for diagnosis and chemotherapy. Endocrine, Metabolic & Immune Disorders Drug Targets, 11, 54–67. 10.2174/187153011794982068 21348820

[acel13075-bib-0026] Seluanov, A. , Hine, C. , Azpurua, J. , Feigenson, M. , Bozzella, M. , Mao, Z. , … Gorbunova, V. (2009). Hypersensitivity to contact inhibition provides a clue to cancer resistance of naked mole‐rat. Proceedings of the National Academy of Sciences of the United States of America, 106, 19352–19357. 10.1073/pnas.0905252106 19858485 PMC2780760

[acel13075-bib-0027] Takada, Y. , Numata, T. , & Mori, Y. (2013). Targeting TRPs in neurodegenerative disorders. Current Topics in Medicinal Chemistry, 13, 322–334. 23432063 10.2174/1568026611313030009

[acel13075-bib-0028] Toth, B. I. , Olah, A. , Szollosi, A. G. , & Biro, T. (2014). TRP channels in the skin. British Journal of Pharmacology, 171, 2568–2581. 10.1111/bph.12569 24372189 PMC4009000

[acel13075-bib-0029] Venkatachalam, K. , & Montell, C. (2007). TRP channels. Annual Review of Biochemistry, 76, 387–417. 10.1146/annurev.biochem.75.103004.142819 PMC4196875 17579562

[acel13075-bib-0030] Watanabe, S. , Kawamoto, S. , Ohtani, N. , & Hara, E. (2017). Impact of senescence‐associated secretory phenotype and its potential as a therapeutic target for senescence‐associated diseases. Cancer Science, 108, 563–569. 10.1111/cas.13184 28165648 PMC5406532

[acel13075-bib-0031] Eischen, C. M. (2016). Genome Stability Requires p53. Cold Spring Harb Perspect Med, 6, a026096. https://doi:10.1101/cshperspect.a026096 10.1101/cshperspect.a026096 PMC4888814 27252396

[acel13075-bib-0032] Yue, Z. , Xie, J. , Yu, A. S. , Stock, J. , Du, J. , & Yue, L. (2015). Role of TRP channels in the cardiovascular system. American Journal of Physiology: Heart and Circulatory Physiology, 308, H157–182. 10.1152/ajpheart.00457.2014 25416190 PMC4312948

