# Supplementary material for: Nociceptive transient receptor potential canonical 7 (TRPC7) mediates aging‐associated tumorigenesis induced by ultraviolet B
Source: Aging Cell. 2019 Nov 21;19(1):e13075. doi: 10.1111/acel.13075 (PMC6974716; doi:10.1111/acel.13075)
Supplement: Supplementary file 1 [file ACEL-19-e13075-s001.docx]

**SUPPORTING INFORMATION**

**Nociceptive transient receptor potential canonical 7 (TRPC7) mediates aging-associated tumorigenesis induced by UVB**


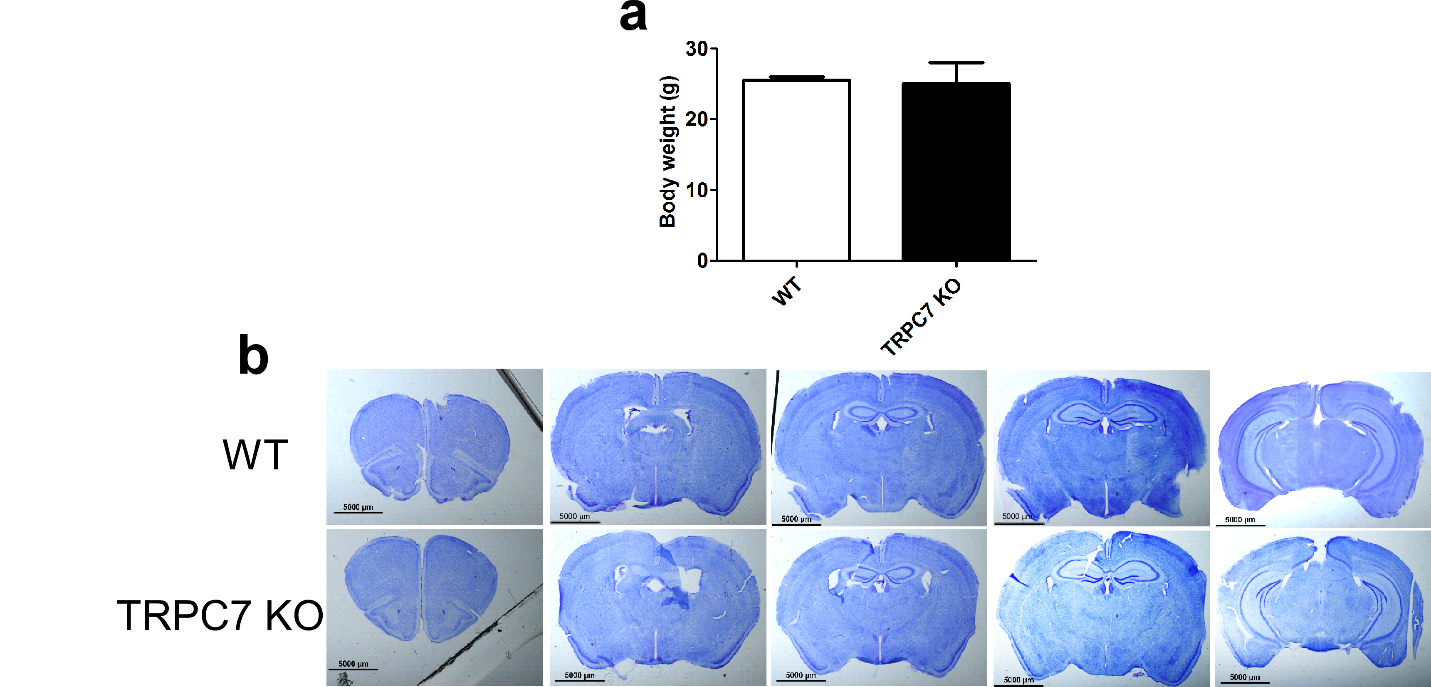


**SUPPORTING INFORMATION FIGURE S1** Generally normal physiology and anatomy in *TRPC7* knockout mice. (a) Comparison of body weight between wild-type (WT) and *TRPC7* knockout (TRPC7 KO) mice. No significant difference was observed. (b) Nissl stains of coronal brain sections from WT and *TRPC7* KO mice. No obvious differences were observed in gross anatomy or neuronal distributions between the brains of WT and *TRPC7* KO mice.

**SUPPORTING INFORMATION TABLE S1** Agonists and antagonists of TRP channels in the skin

| **TRP** | **Agonist** | **Antagonist** |
| --- | --- | --- |
| C1 | PLC pathway | Gd, La, SKF96365, 2-APB, Na DNA |
| C4 | La^3+^ (100 μM), calmidazolium, PLC  pathway | 2-APB, niflumic acid, DIDS, La (mM),  SKF96365, Na DNA |
| C5 | Sphingosine-1-phosphate, La^3+^ (100  μM), PLC pathway, nitric oxide | 2-APB, flufenamate, La (mM),  SKF96365, Na DNA |
| C6 | DAG, 20-HETE, PIP3, PLC  pathway | 2-APB, amiloride, Cd, La, Gd,  SKF96365, Na DNA |
| C7 | DAG, PLC pathway | 2-APB, flufenamate, La, SKF96365, Na  DNA |
| V1 | H^+^, PLC pathway, anandamide,  2-AG, 20-HETE, HPETE,  capsaicin, olvanil, resiniferatoxin,  arvanil, piperine, OEA, 2-APB,  DPBA, camphor, DAG, ethanol,  nicotine, proinflammatory  cytokines, PIP2 | Capsazepine, RuR, acylpolyamines,  camphor (desensitizes), SKF96365 |
| V3 | T>30°C, 2-APB, DPBA, carvacrol,  thymol, eugenol, camphor,  ethanol, vanillin, menthol,  cinnemaldehyde, PUFAs, cloves,  thyme | RuR, La |
| V6 | PIP2, Ca^2+^ | RuR, Cd>>Gd>La, Mg |
| M2 | ADP ribose, cADP ribose, βNAD,  H_2_O_2_, hypo-osmolarity  (enhances), arachidonic acid, Ca^2+^ | ADP, econazole, miconazole,  clotrimazole, flufenamate |
| M8 | T<25°C, PIP2, menthol, icilin,  eucalyptol | BCTC, capsazepine, 2-APB |
| A1 | Icilin, isothiocyanates, allicin,  cinnamaldehyde, acrolein,  cannabinoids, bradykinin, DAG,  PUFAs, (-)-trans-Δ 9-THC,  carvacrol, eugenol, gingerol | RuR, amiloride, camphor, menthol,  gentamicin, Gd, carvacrol  (desensitizes), GRC17536 |

ADP, adenosine 5’-diphosphate; 2-AG, 2-arachidonyl glycerol; 2-APB, 2-aminoethoxydiphenyl borate; BCTC, (N-(4-tertiarybutylphenyl)-4-(3-chloropyridin-2-yl)tetrahydropyrazine-1(2H)-carbox-amide); cADP, adenosine 3’,5’-cyclic diphosphate; DAG, diacylglycerol; DIDS, 4,4’-diisothiocyanatostilbene-2,2’-disulphonic acid; DPBA, diphenylboronic anhydride; 20-HETE, 20-hydroxy-(5,8,11,14)-eicosatetraenoic acid; H_2_O_2_, hydrogen peroxide; HPETE, hydroperoxyeicosatetraenoic acid; Na DNA, deoxyribonucleic acid sodium salt; βNAD, β-nicotinamide adenine dinucleotide; OEA, oleoylethanolamide; PIP2, phosphatidylinositol (4,5)-bisphosphate; PIP3, phosphatidylinositol (3,4,5)-trisphosphate; PLC, phospholipase C; PUFAs, polyunsaturated fatty acids; RuR, ruthenium red; (-)-trans-Δ 9-THC, (-) trans-delta-9-tetrahydrocannabinol(Caterina *et al.,* 1997; Clapham 2007; Venkatachalam & Montell 2007; Acharya *et al.,* 2013; Hsu *et al.,* 2015).


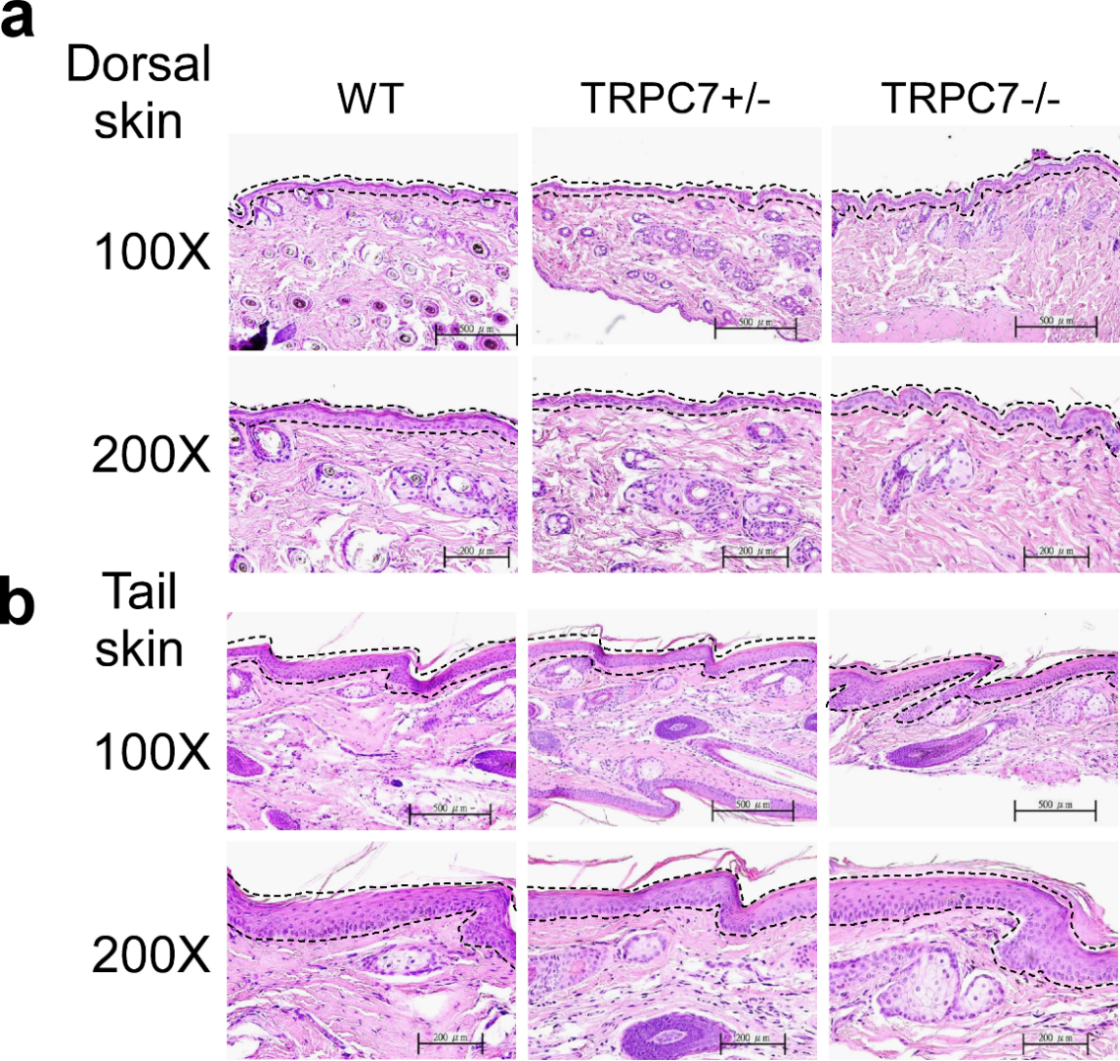


**SUPPORTING INFORMATION FIGURE S2** No difference in skin composition between wild-type (WT) and *TRPC7* knockout mice. After hair removal for 8 days, (a) dorsal skin and (b) tail skin of WT (*n* = 3), *TRPC7+/-* (*n* = 3), and *TRPC7-/-* (*n*=3) mice were punched and sectioned for staining with hematoxylin and eosin. Dotted lines indicate the boundaries of the epidermis.


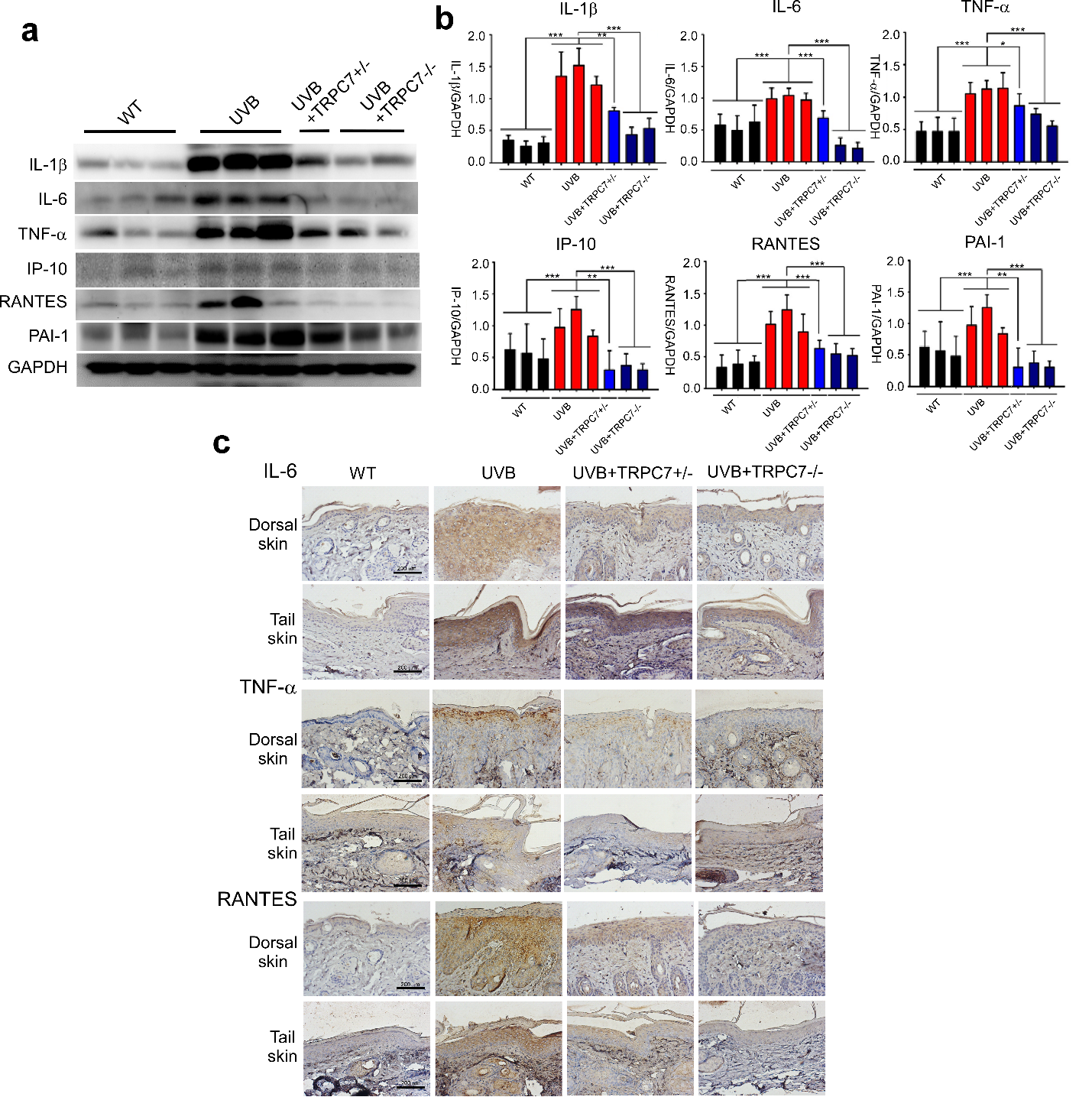


**SUPPORTING INFORMATION FIGURE S3** TRPC7 deficiency downregulated the senescence-associated secretory phenotype (SASP) in UVB-induced epidermal aging. (a) Western blot analysis of SASP proteins IL-1β, IL-6, TNF-α, IP-10, RANTES, and PAI-1 in wild-type (WT) and *TRPC7* knockout mice (*TRPC7+/-* and *TRPC7-/-*). (b) Quantification of the mean levels (± standard deviation) of SASP proteins shown in (a). **P* < 0.05; ***P* < 0.01; ****P* < 0.001. (c) Immunohistochemical staining showing that the expression of SASP proteins IL-6, TNF-α, and RANTES (brown, counterstained with hematoxylin) was decreased in the skin of *TRPC7-/-* mice.


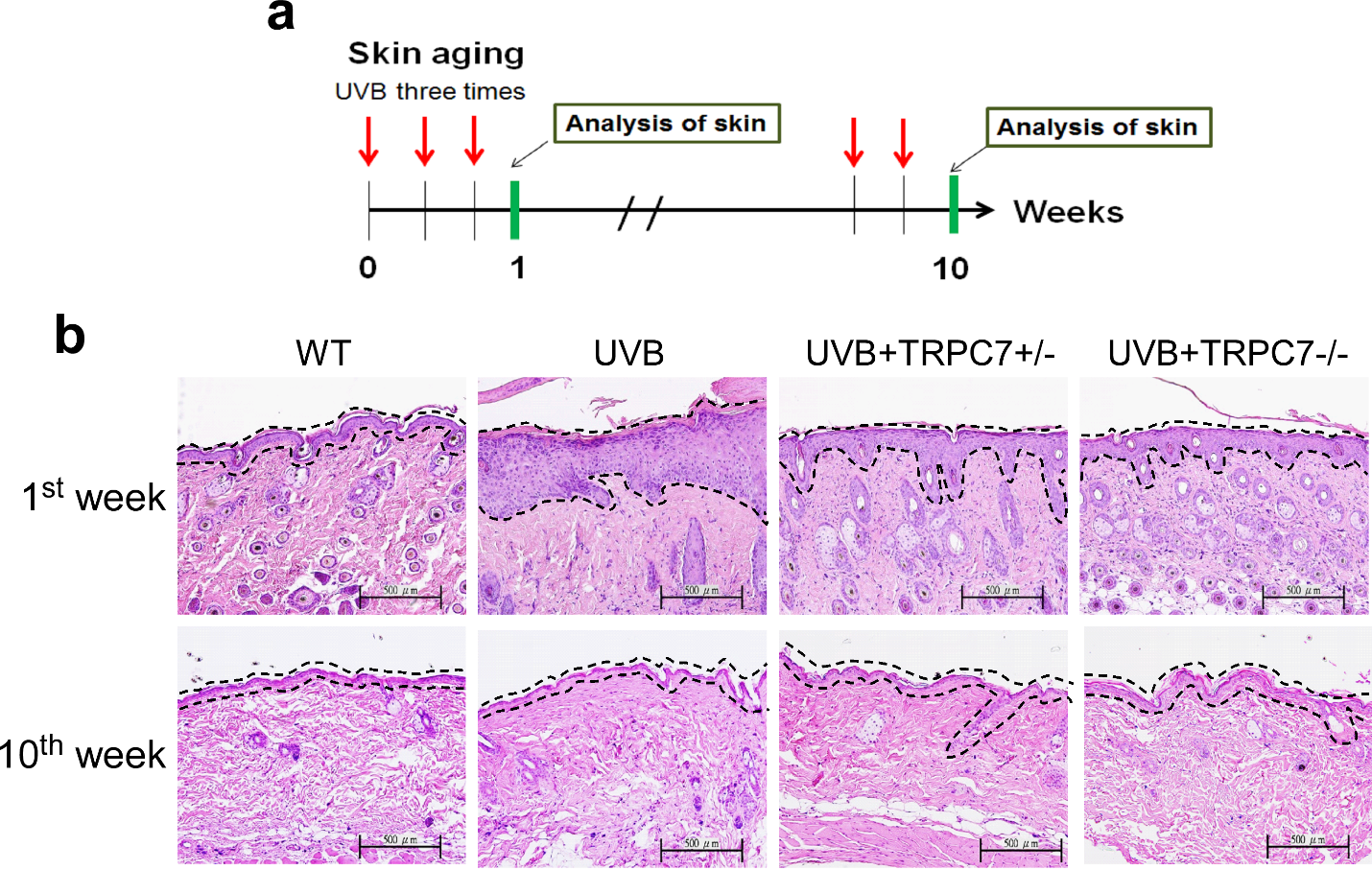


**SUPPORTING INFORMATION FIGURE S4** Recovery of the epidermis to a single layer over time in UVB-irradiated dorsal skin. (a) The experimental design for examining the effects of ultraviolet B (UVB) exposure (administered every 2 days for 10 weeks) on the epidermis of wild-type (WT) and *TRPC7* knockout mice (*TRPC7+/-* and *TRPC7-/-*). (b) Hematoxylin and eosin–stained sections showing the epidermis of UVB-irradiated dorsal skin. The boundaries of the epidermis are shown by the dotted lines. Dorsal skin was punched after UVB exposure for 1 week or 10 weeks and was sectioned and stained.


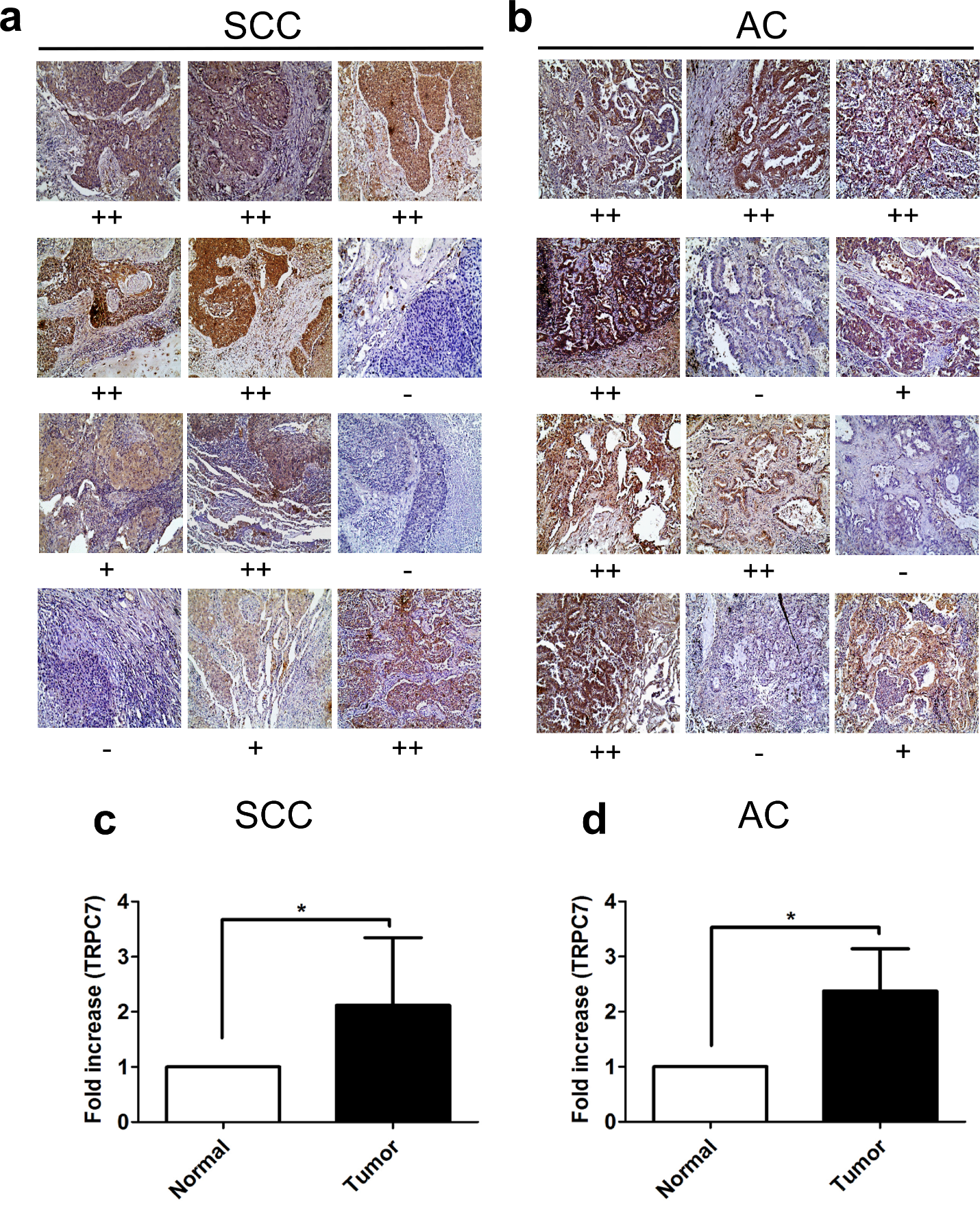


**SUPPORTING INFORMATION FIGURE S5** Overexpression of TRPC7 in tumor biopsies from patients with non-small cell lung cancer (NSCLC). NSCLC is the most common type of lung cancer (about 85% of lung cancers); the two major types of NSCLC are squamous cell carcinoma (SCC) and adenocarcinoma (AC). TRPC7 expression in (a) SCC and (b) AC was detected by using immunohistochemistry. ++ indicates high protein expression, and + or – indicates moderate or low protein expression, respectively. Quantification of the mean (± standard deviation) expression of TRPC7 in (c) SCC and (d) AC. **P* < 0.05.

**SUPPORTING INFORMATION TABLE S2** Correlation between clinical pathologic features and *TRPC7* gene expression in non-small cell lung cancer (NSCLC) patients

|  | Squamous cell carcinoma (n=38) | | |  |  |  | Adenocarcinoma (n=43) | | |  |
| --- | --- | --- | --- | --- | --- | --- | --- | --- | --- | --- |
|  |  |  |  |  |  |  |  | | |  |
| Characteristics |  | **Low** | **High** | **P value** |  | **Characteristics** |  | **Low** | **High** | **P value** |
|  | n | n (%) | n (%) |  |  |  | n | n (%) | n (%) |  |
| Sex |  |  |  |  |  | Sex |  |  |  |  |
| Women | 10 | 2 (20) | 8 (89) |  |  | Women | 24 | 9 (37.5) | 15 (62.5) |  |
| Men | 28 | 9 (32.1) | 19 (67.9) | 0.69 |  | Men | 19 | 5 (26.3) | 14 (73.7) | 0.653 |
| Age (years) | |  |  |  |  | Age (years) | |  |  |  |
| < 60 | 3 | 1 (33.3) | 2 (66.7) |  |  | < 60 | 10 | 4 (40) | 6 (60) |  |
| ≥ 60 | 35 | 10 (28.6) | 25 (71.4) | 1 |  | **≥** 60 | 33 | 10 (30.3) | 23 (69.7) | 0.7037 |
| Tumor size (cm) | |  |  |  |  | Tumor size (cm) | |  |  |  |
| < 3 | 12 | 8 (66.7) | 4 (33.3) |  |  | < 3 | 19 | 11 (57.9) | 8 (42.1) |  |
| ≥ 3 | 26 | 3 (11.5) | 23 (88.4) | 0.0011^*^ |  | ≥ 3 | 24 | 3 (12.5) | 21 (87 .5) | 0.0028^*^ |
| Stage (UICC) | |  |  |  |  | Stage (UICC) | |  |  |  |
| 1 + 2 | 28 | 9 (32.1) | 19 (67.9) |  |  | 1 + 2 | 31 | 12 (38.7) | 19 (61.3) |  |
| 3 + 4 | 10 | 2 (20) | 8 (80) | 0.69 |  | 3 + 4 | 12 | 2 (16.7) | 10 (83.3) | 0.2785 |
| Histology |  |  |  |  |  | Histology |  |  |  |  |
| WD + MD | 24 | 6 (25) | 18 (75) |  |  | WD + MD | 34 | 11 (32.4) | 23 (67.6) |  |
| PD + UD | 14 | 5 (32.7) | 9(62.3) | 0.7121 |  | PD + UD | 9 | 3 (33.3) | 6 (66.7) | 1 |
| Vascular invasion | |  |  |  |  | Vascular invasion | |  |  |  |
| Negative | 21 | 9 (42.9) | 12 (57.1) |  |  | Negative | 30 | 11 (36.7) | 19 (63.3) |  |
| Positive | 17 | 2 (11.8) | 15 (88.2) | 0.072 |  | Positive | 13 | 3 (23.1) | 10 (76.9) | 0.491 |
| Perineural invasion | |  |  |  |  |  |  |  |  |  |
| Negative | 28 | 7 (25) | 21 (75) |  |  |  |  |  |  |  |
| Positive | 10 | 4 (40) | 6 (60) | 0.4318 |  |  |  |  |  |  |

MD, moderately well differentiated; PD, poorly differentiated; UD, undifferentiated; UICC, Union for International Cancer Control; WD, well differentiated.

**P* < 0.01, indicated in bold.

**REFERENCES**

Acharya SA, Portman A, Salazar CS, Schmidt JJ (2013) Hydrogel-stabilized droplet bilayers for high speed solution exchange. *Sci Rep*. **3**, 31a39.

Caterina MJ, Schumacher MA, Tominaga M, Rosen TA, Levine JD, Julius D (1997) The capsaicin receptor: a heat-activated ion channel in the pain pathway. *Nature*. **389**, 816-824.

Clapham DE (2007) SnapShot: mammalian TRP channels. *Cell*. **129**, 220.

Hsu WL, Lu JH, Noda M, Wu CY, Liu JD, Sakakibara M, Tsai MH, Yu HS, Lin MW, Huang YB, Yan SJ, Yoshioka T (2015) Derinat Protects Skin against Ultraviolet-B (UVB)-Induced Cellular Damage. *Molecules*. **20**, 20297-20311.

Venkatachalam K, Montell C (2007) TRP channels. *Annu Rev Biochem*. **76**, 387-417.
